# Heliciopsides A−E, Unusual Macrocyclic and Phenolic Glycosides from the Leaves of *Heliciopsis terminalis* and Their Stimulation of Glucose Uptake

**DOI:** 10.3390/ph15111315

**Published:** 2022-10-25

**Authors:** Byeol Ryu, Eun-Jin Park, Thi-Phuong Doan, Hyo-Moon Cho, Jin-Pyo An, Thi-Linh-Giang Pham, Ha-Thanh-Tung Pham, Won-Keun Oh

**Affiliations:** 1 Korea Bioactive Natural Material Bank, Research Institute of Pharmaceutical Sciences, College of Pharmacy, Seoul National University, Seoul 08826, Korea; 2 Department of Botany, Hanoi University of Pharmacy, Hanoi 000084, Vietnam

**Keywords:** *Heliciopsis terminalis*, Proteaceae, macrocyclic glycosides, biphenyl glycoside, glucose uptake, AMPK

## Abstract

Ten phenolic constituents, including three new macrocyclic glycosides (**1**–**3**), a new phenolic glycoside (**5**), a new biphenyl glycoside (**6**), and five known compounds (**4**, **7**–**10**), were isolated from a 70% MeOH extract of the leaves of *Heliciopsis terminalis* by liquid chromatography-tandem mass spectrometry (LC-MS/MS)-guided molecular networking. The chemical structures of new compounds **1**–**3**, **5** and **6** were established based on comprehensive spectroscopic data analysis, including 1D and 2D NMR and HRESIMS techniques. All isolated compounds (**1**–**10**) were evaluated for their stimulation of glucose uptake in differentiated 3T3-L1 adipocytes using 2-deoxy-2-[(7-nitro-2,1,3-benzoxadiazol-4-yl)amino]-d-glucose (2-NBDG) as a fluorescent glucose analog. Compounds **3**, **6** and **8** showed stimulatory effects on the uptake of 2-NBDG in 3T3-L1 adipocyte cells. Among them, compounds **3** and **6** activated the AMPK signaling pathway in differentiated C2C12 myoblasts.

## 1. Introduction

Diabetes mellitus (DM) is a chronic metabolic disease that causes abnormally high blood glucose levels. DM is typically divided into types 1 and 2, with people predominantly suffering from type 2 diabetes mellitus (T2DM), which mainly involves the inoperability of produced insulin at the site of action [1]. This eventually leads to hyperglycemia, insulin resistance, and an imbalance in insulin secretion, and if disease progression is severe, diabetes-related complications can occur in the heart, kidneys, blood vessels, eyes, and nerves [2]. The rapid increase in the number of patients with diabetes has long been a global public health concern, and modern society has continued to pay attention to the development of antidiabetic drugs without side effects. Glucose transporter 4 (GLUT4) is an important protein involved in the pathogenesis of T2DM. The impaired translocation or reduced expression of GLUT4 attenuates glucose uptake in skeletal muscles and adipose tissues and causes severe insulin resistance and glucose intolerance [3,4,5,6]. Increasing glucose uptake into peripheral tissues such as skeletal muscle or adipose tissue is an important strategy to remove glucose from the blood for T2DM management [7]. AMPK activation in skeletal muscle promotes glucose transport by enhancing GLUT4 translocation in response to insulin. Thus, stimulation of AMPK activity is an interesting therapeutic strategy to lower blood glucose levels in T2DM [8,9,10,11]. Therefore, our group has continued its research to discover insulin-mimetic agents in natural products, focusing on the aforementioned mechanisms [12,13,14,15,16].

The evergreen tree *Heliciopsis terminalis* (Kurz) Sleum (www.theplantlist.org) (synonyms, *Helicia terminalis* Kurz or *Heliciopsis lobata* var. *microcarpa* C.Y. Wu & T.Z. Hsu) is native to Cambodia, East Himalaya, Laos, Myanmar, Thailand, and Vietnam and grows to approximately 7–8 m high [17]. In Vietnam, native people have used *H. terminalis* to treat rheumatism and several other diseases, and a remedy consisting of the trunk of *H. terminalis*, *Gynostemma pentaphyllum*, and *Solanum procumbens* has been prescribed to prevent liver disorder [18]. *H. terminalis* is one of 12 plants that make up the extract Prasachandaeng (PSD), which has been used as a traditional antipyretic medicine in Thailand [19]. Recent studies on the trunk of *H. terminalis* demonstrated the biological activities of bisresorcinol isolated from this plant [20]. However, to date, phytochemical and biological investigations of the constituents of *H. terminalis* have not been conducted, with the exception of bisresorcinol. Thus, research on various chemical components and biological activities of *H. terminalis* is needed. As part of ongoing research to find antidiabetic agents [12,13,14,15,16,21], MS/MS-molecular networking was applied to investigate the chemical diversity of *H. terminalis* leaves. Compounds isolated from the leaves of *H. terminalis* were evaluated for their potential to stimulate 2-deoxy-2-[(7-nitro-2,1,3-benzoxadiazol-4-yl)amino]-d-glucose (2-NBDG) uptake in differentiated adipocytes, and the studies on the mechanism were followed. In this study, we present phenolic constituents in the leaves of *H. terminalis* as a candidate for antidiabetic agents.

## 2. Results and Discussion

### 2.1. Structure Elucidation

**Figure 1:**
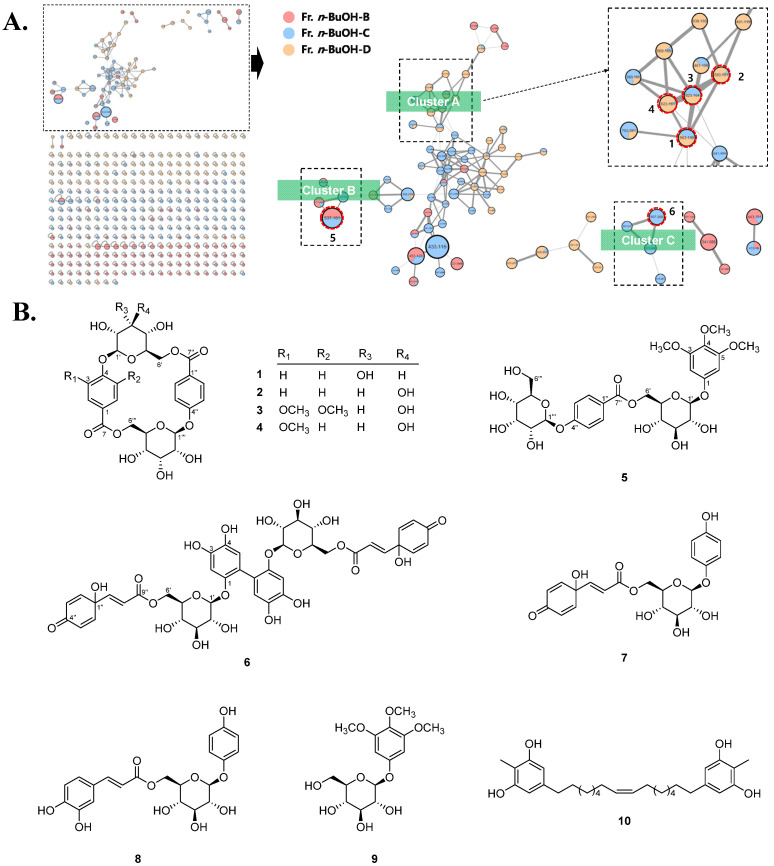
(**A**) LC-MS/MS molecular networking for the subfractions of the *n*-BuOH layer, and (**B**) chemical structures of compounds **1**–**10** isolated from the leaves of *H. terminalis*.

To profile the secondary metabolites of the leaves of *H. terminalis*, the 70% MeOH extract was partitioned with *n*-hexane, EtOAc, *n*-BuOH, and water and subjected to LC-MS/MS analysis (Figure S1). In the LC-MS/MS chromatogram of the 70% MeOH extract, the majority of metabolites from the leaves of *H. terminalis* were detected in the polar region (t_R_ of 0.00–5.00 min, 10%–30% MeCN), and these polar constituents were mostly located in the EtOAc and *n*-BuOH layers. In an effort to discover new metabolites, LC-MS/MS-based molecular networking (MN) was applied to subfractions B.B–B.D obtained from the *n*-BuOH layer (Figure 1A). As a result, macrocyclic glycosides (**1**–**4**) and phenolic glycoside (**5**) were identified from clusters A and B, respectively. Furthermore, one of the nodes (*m*/*z* 897.21) in cluster C possessing a high molecular weight (approximately 900 Da), was identified as the symmetric biphenyl glycoside (**2**), which has rarely been reported in nature. The known compounds were identified as clemochinenoside D (**4**) [22], robustaside D (**7**) [23], robustaside B (**8**) [23], 3,4,5-trimethoxyphenyl-*β*-d-glucopyranoside (**9**) [24], and kusukuenol B_1_ (**10**) [25] by comparison of the spectroscopic data with those in the literature.

**Figure 2:**
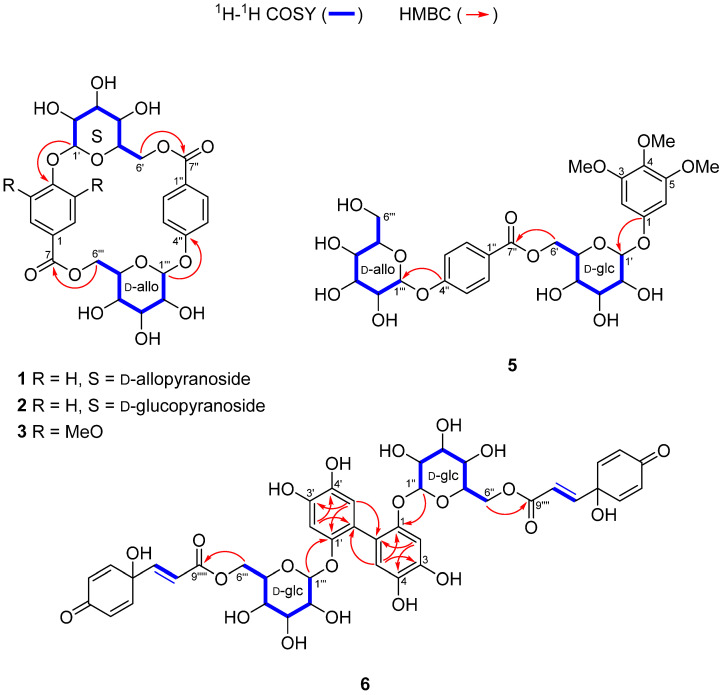
Key ^1^H−^1^H COSY and HMBC correlations for compounds **1**–**3**, **5** and **6**.

**Figure 3:**
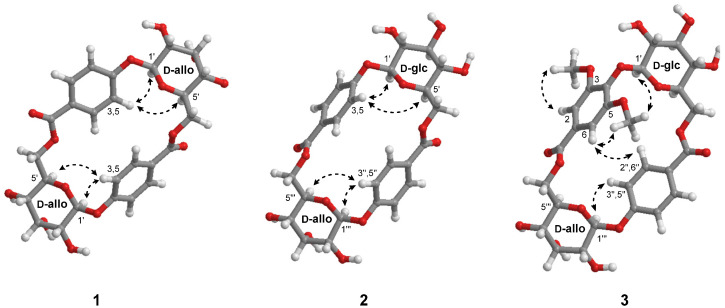
Key ROESY correlations for compounds **1**–**3**.

Compound **1** was obtained as a white amorphous powder. The molecular formula was determined to be C_26_H_28_O_14_ based on the [M–H]^−^ peak at *m*/*z* 563.1399 (calcd for C_26_H_27_O_14_, 563.1401) by HRESIMS, indicating 13 degrees of unsaturation. The ^1^H and ^13^C NMR spectra of **1** (Table 1) exhibited the typical signals of a 4-hydroxybenzoyl group [*δ*_H_ 8.02 (d, *J* = 8.8 Hz, H-2, 6, 2H) and *δ*_H_ 7.19 (d, *J* = 8.8 Hz, H-3, 5, 2H); *δ*_C_ 122.4 (C-1), 131.3 (C-2, 6), 115.8 (C-3, 5), 160.6 (C-4), 165.1 (C-7)]. The remaining six carbon signals were determined to be from the hexapyranosyl sugar unit by analysis of the ^1^H−^1^H COSY correlations of H-1′/H-2′/H-3′/H-4′/H-5′/H-6′, and the assigned carbon signals of the hexapyranosyl moiety [*δ*_C_ 96.8 (C-1′), 69.9 (C-2′), 71.5 (C-3′), 68.3 (C-4′), 71.2 (C-5′) and 65.4 (C-6′)] are consistent with the reported ^13^C NMR chemical shifts of allopyranoside (Figure 2 and Table 1) [26,27,28]. The anomeric proton signal with a large coupling constant at *δ*_H_ 5.39 (d, *J* = 8.0 Hz, H-1′) suggested that the allopyranosyl moiety was in the *β*-configuration. The linkage between the *β*-allopyranosyl moiety and the 4-hydroxybenzoyl unit was established by HMBC correlations from H-1″ to C-4 (Figure 2). Only 13 carbon signals were displayed in the ^13^C NMR spectrum of **1**. However, the HRESIMS data indicated that compound **1** possessed 26 carbons, which means that **1** is symmetric. Finally, the HMBC correlation from H-6a′/H-6b′ to C-7″ suggested that **1** was a symmetric macrocyclic glycoside (Figure 2). The ROESY correlations of H-3 and H-5 with H-1′ and H-5′ further confirmed that **1** was a macrocyclic glycoside (Figure 3). Additionally, the sugar moiety of **1** was determined to be d-allopyranoside through derivatization of the afforded sugar by acid hydrolysis of **1** and comparison of the *t*_R_ with the chiral derivative of the authentic sample [29]. Taken together, the structure of **1** was elucidated as a 4-[(6′-*O*-*β*-d-allopyranosyl)oxy]hydroxy-benzoic acid cyclic dimeric inner ester and was named heliciopside A.

Compound **2** was obtained as a white amorphous powder, and it had the same molecular formula as that of 1 (C_26_H_28_O_14_) as deduced from the [M–H]^−^ peak at *m*/*z* 563.1403 by HRESIMS (calcd for C_26_H_27_O_14_, 563.1401). The ^1^H and ^13^C NMR spectra of 2 (Table 1) exhibited two 4-hydroxybenzoyl group signals at *δ*_H_ 7.99 (d, *J* = 8.8 Hz, H-2, 6, 2H), 7.19 (d, *J* = 8.8 Hz, H-3, 5, 2H), 7.98 (d, *J* = 8.8 Hz, H-2″, 6″, 2H), and 7.18 (d, *J* = 8.8 Hz, H-3″, 5″, 2H) with carbon signals at *δ*_C_ 122.6 (C-1), 131.2 (C-2, 6), 115.8 (C-3, 5), 160.4 (C-4), 165.0 (C-7) and *δ*_C_ 122.5 (C-1″), 131.2 (C-2″, 6″), 115.8 (C-3″, 5″), 160.4 (C-4″), and 165.0 (C-7″) (Table 1). Additionally, two *β*-configured anomeric proton signals at *δ*_H_ 5.22 (d, *J* = 7.2 Hz, H-1′) and *δ*_H_ 5.37 (d, *J* = 8.0 Hz, H-1‴) appeared. Similar to compound **1**, two 4-hydroxybenzoyl units and two hexapyranosyl moieties were found in the ^1^H and ^13^C NMR spectra of **2**. However, the 26 carbon signals in the ^13^C NMR spectrum of **2** indicated that compound **2** is asymmetric, which means that the two hexapyranosyl groups should be different. The ^1^H−^1^H COSY analysis revealed that the two sets of hexapyranosyl signals in the ^1^H and ^13^C NMR spectra were *β*-allopyranoside and *β*-glucopyranoside (Figure 2 and Table 1). These results were further confirmed by comparison of the NMR chemical shifts with those reported [26,27,30]. The two sugar units were finally determined to be in the d-form through derivatization of the afforded sugar by acid hydrolysis of **2** [29]. The linkages of the two 4-hydroxybenzoyl groups, *β*-d-allopyranoside, and *β*-d-glucopyranoside were established by the observed HMBC correlations from H-1″ to C-4, from H-1‴ to C-4″, from H-6a′/H-6b′ to C-7″, and from H-6a″/H-6b″ to C-7″ (Figure 2). In the ROESY spectrum, cross-peaks of H-3, 5 with H-1′ and H-5′ and H-3″, 5″ with H-1‴ and H-5‴ appeared, indicating that 2 is a macrocyclic glycoside (Figure 3). Thus, the structure of **2** was elucidated as 4-({6′-*O*-[(4′′-*O*-*β*-d-allopyranosyl)hydroxy-benzoyl]-*β*-d-glucopyranosyl}oxy)hydroxyl-benzoic acid inner ester and named as heliciopside B.

Compound **3** was purified as a white amorphous powder and had a molecular formula of C_28_H_32_O_16_ determined by HRESIMS (*m*/*z* 623.1608 [M–H]^−^, calcd for C_28_H_31_O_16_, 623.1612). From the difference in the molecular formula between compounds **1** (or **2**) and **3**, the structure of compound **3** was inferred to contain two methoxy groups more than compound **1** (or **2**). These results were evidenced by additional 1D NMR signals at *δ*_H_ 3.61 (*δ*_C_ 56.0, 3-OMe) and *δ*_H_ 3.98 (*δ*_C_ 56.5, 5-OMe) (Table 1). Assignments of ^1^H and ^13^C NMR for 3 (Table 1) by 1D NMR, HSQC, and HMBC spectral analysis exhibited that 3 consisted of a 4-hydroxybenzoyl group, a syringoyl group, and two hexapyranosyl groups. Additionally, the two hexapyranosyl moieties were determined to be d-allopyranoside and d-glucopyranoside by ^1^H–^1^H COSY analysis and derivatization of the sugar afforded from acid hydrolysis [29]. Furthermore, two anomeric proton signals at *δ*_H_ 5.36 (d, *J* = 7.2 Hz, H-1′) and *δ*_H_ 5.17 (d, *J* = 8.0 Hz, H-1‴) displayed a large coupling constant, suggesting that both sugar units were *β*-configured. The HMBC correlations from H-1′ to C-4, from H-1‴ to C-4″, from H-6′ to C-7″, and from H-6‴ to C-7 suggested the linkages of two sugars, the 4-hydroxybenzoyl unit, and the syringoyl unit. The ROE cross-peaks of MeO-5 with H-1′ and H-1‴ and H-5‴ with H-3″, 5″ in the ROESY spectrum showed that compound **3** is a macrocyclic glycoside. Notably, 3 possesses 4-hydroxybenzoyl and syringoyl groups, both of which are individually symmetric structures, but the ^13^C NMR signals for the 4-hydroxybenzoyl group [*δ*_C_ 122.5 (C-1″), 130.7 (C-2″, 6″), 115.9 (C-3″, 5″), 160.7 (C-4″), 164.9 (C-7″)] were revealed to be symmetric, whereas those for the syringoyl group [*δ*_C_ 124.6 (C-1), 107.0 (C-2), 153.1 (C-3), 137.7 (C-4), 152.0 (C-5), 106.7 (C-6), 164.9 (C-7)] were not. Through these observations, it was inferred that the 4-hydroxybenzoyl unit could rotate freely, whereas the syringoyl unit has restricted mobility in the structure due to the large 3,5-OMe substituents [26]. Furthermore, the orientation of the syringoyl unit was determined as MeO-5 and H-6 oriented inside, and MeO-3 and H-2 oriented opposite by the ROESY cross-peaks of H-6 and H-2″, 6″. Therefore, the structure of **3** was elucidated as 4-({6′-*O*-[(4′′-*O*-*β*-d-allopyranosyl)hydroxy-benzoyl]-*β*-d-glucopyranosyl}oxy)syringic acid inner ester and was named as heliciopside C. 

Compound **5** was obtained as a white amorphous powder and had a molecular formula of C_28_H_36_O_16_ as determined by HRESIMS (*m*/*z* 627.1925 [M–H]^−^, calcd for C_28_H_35_O_16_, 627.1925). The ^1^H NMR spectrum (Table 1) of **5** showed a symmetrical 1,3,4,5-tetrasubstituted benzene ring at *δ*_H_ 6.32 (s, 2H, H-2, 6) with three methoxy groups at *δ*_H_ 3.65 (s, 6H, MeO-3, 5) and 3.57 (s, 3H, MeO-4), a 4-hydroxy benzoyl group at *δ*_H_ 7.89 (d, *J* = 8.5 Hz, H-2″, 6″) and *δ*_H_ 7.09 (d, *J* = 8.5 Hz, H-3″, 5″) and two *β*-configured anomeric protons at *δ*_H_ 5.23 (d, *J* = 7.5 Hz, H-1‴) and *δ*_H_ 4.98 (d, *J* = 8.0 Hz, H-1′). ^1^H NMR signals for two hexapyranosyl groups were determined by ^1^H−^1^H COSY analysis (Figure 2), and the sugars were determined to be d-glucopyranoside and d-allopyranoside [29]. The connectivity of the four substructures was determined by HMBC correlations from H-1″″ to C-4″, from H-6′ to C-7″, and from H-1′ to C-1. Finally, compound **5** was deduced as 3,4,5-trimethoxyphenyl-4-*O*-[6-*O*-(4-*O*-*β*-d-allopyranosyl)-hydroxy-benzoyl]-*β*-d-glucopyranoside and was named as heliciopside D.

Compound **6** was purified as a brown amorphous solid, and its molecular formula was deduced as C_42_H_42_O_22_ by HRESIMS analysis (*m*/*z* 897.2102 [M–H]^−^, calcd for C_42_H_41_O_22_, 897.2089), indicating 22 degrees of unsaturation. In the ^1^H NMR spectrum, two AB systems of aromatic protons at *δ*_H_ 6.89 (m, H-2″, 6″) and *δ*_H_ 6.22 (m, H-3″, 5″) and trans-olefinic protons at *δ*_H_ 6.72 (d, *J* = 16.2 Hz, H-7″) and *δ*_H_ 6.30 (d, *J* = 16.2 Hz, H-8″) were appeared (Table 2). The ^13^C NMR spectrum exhibited a conjugated ketone at *δ*c 187.2 (C-4″), a conjugated ester at *δ*c 167.4 (C-9″), and an oxygenated quaternary carbon at *δ*c 70.4 (C-1″). Thus, it was confirmed that the (*E*)-3-(1-hydroxy-4-oxocyclohexa-2,5-dien-1-yl) acrylate moiety was present in compound **6**. The 1D NMR and ^1^H–^1^H COSY interpretation of compound **6** (Table 2 and Figure 2) suggested the presence of a *β*-glucopyranoside unit in its d-form [29]. The 1,3,5,6-tetrasubstituted benzene ring was also deduced by the ^1^H NMR signals of two singlet aromatic protons at *δ*_H_ 6.66 (s, H-2) and *δ*_H_ 6.62 (s, H-5) together with the ^13^C NMR signals of three oxygenated quaternary carbons (*δ*c 148.8, 145.9, and 141.5), two protonated aromatic carbons (*δ*c 119.1 and 106.7), and a quaternary carbon (*δ*c 121.4) in the aromatic region of the ^13^C NMR spectrum. The linkage between the three subunits was determined by HMBC correlations from H-1′ to C-1 and from H-6′a/ 6′b to C-9″. From these results, compound **6** was found to have a structure similar to that of robustaside D, except that the two protonated aromatic carbons of robustaside D were replaced by one oxygenated aromatic carbon and one quaternary aromatic carbon [23]. However, as there were 21 carbon signals in the ^13^C NMR spectrum of compound **6**, and its molecular formula indicates that it has 42 carbons, these results suggested that compound **6** is a symmetric dimer. Finally, detailed HMBC analysis determined that the biphenyl group in compound **6** is linked via a C-C bond between C-4/C-4 (Figure 2). Taken together, compound **6** consists of two (*E*)-3-(1-hydroxy-4-oxocyclohexa-2,5-dien-1-yl)acrylate units, two *β*-d-glucopyranosides, and a hexasubstituted biphenyl group as a unique symmetric structure. Thus, compound **6** was deduced as 2′-[[6-*O*-[(2*E*)-3-(1-hydroxy-4-oxo-2,5-cyclohexadien-1-yl)-2-propen-1-yl]-*β*-d-glucopyranosyl]oxy]-4,4′,5,5′-tetrahydroxy [1,1′-biphenyl]-2-yl,6-[(2*E*)-3-(1-hydroxy-4-oxo-2,5-cyclohexadien-1-yl)-2-propenoate]-*β*-d-glucopyranoside and was named as heliciopside E.

### 2.2. Stimulation of Glucose Uptake

**Figure 4:**
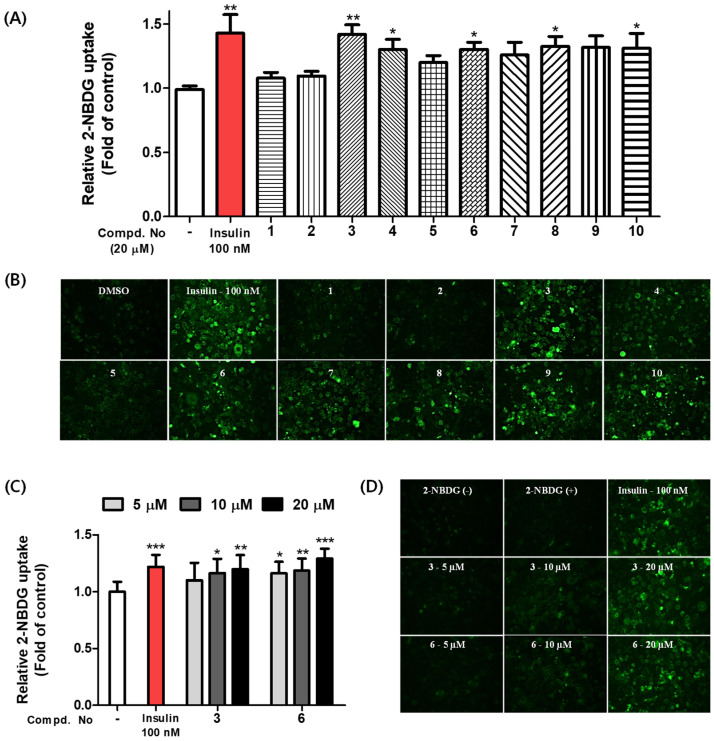
Stimulatory effects of compounds **1**–**10** on glucose uptake in 3T3-L1 adipocytes using a 2-NBDG. (**A**) 3T3-L1 adipocytes were incubated with insulin (100 nM) as a positive control and compounds **1**–**10** (20 μM) for 1 h with or without 2-NBDG. Glucose uptake was measured at Ex/Em = 450/535 nm using a fluorescence microplate reader. The results are presented as the means ± SDs (*n* = 3); each experiment was performed in triplicate. * *p* < 0.05, ** *p* < 0.01, and *** *p* < 0.001, compared to the negative control. (**B**) After differentiated cells were treated with 100 nM insulin and 20 μM test compounds after treatment for 1h at the presence or absence of 2-NBDG, each cell image was obtained with a fluorescence microscope. (**C**,**D**) Concentration−dependent effects of compounds **3** and **6** on glucose uptake in 3T3-L1 adipocytes. Cells were treated with these compounds at 10 and 20 μM or with 100 nM insulin. After 1 h of incubation, the fluorescence intensities images were obtained using fluorescence microscopy.

Compounds **1**–**10** were evaluated for their stimulatory effects on glucose uptake by 2-NBDG glucose uptake assay. 2-NBDG is a fluorescent-tagged glucose analog that is used to observe intracellular glucose uptake. Fully differentiated 3T3-L1 adipocytes, which are sensitive to insulin, were used for this experiment. The transportation efficacy of 2-NBDG into cells was determined by detecting the fluorescence signal in the differentiated adipocytes after treatment with each isolate at a concentration of 20 μM and insulin as a positive control. The green fluorescence increased after treatment with most of the tested compounds, except **1**, **2**, and **5**, compared to the control group (Figure 4A,B and Figure S47). Additionally, 20 μM compounds **1**–**9** exhibited no cytotoxic effects on 3T3-L1 cells as determined by MTT assay (Figure S49). Additional MTT experiments were performed with three different concentrations (5, 10, and 20 µM) of **10** with 3T3-L1 adipocytes (Figure S50). Among the tested compounds, compounds **3** and **6**, which showed strong antidiabetic effects, were further tested for their effects on glucose uptake at various concentrations (5, 10, and 20 μM), and their activities were dose-dependent (Figure 4C,D and Figure S48).

### 2.3. Compounds **3** and **6** Increased Glucose Uptake by Activating the AMPK Signaling Pathway in Differentiated C2C12 Myoblasts

**Figure 5:**
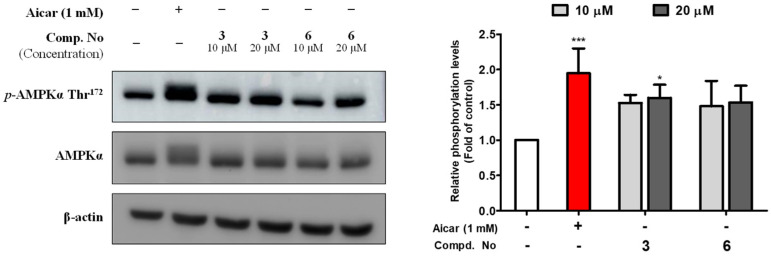
The effects of compounds **3** and **6** on the phosphorylation of the protein AMPK in differentiated C2C12 skeletal myoblasts. C2C12 cells were exposed to compounds **3** and **6** (10 and 20 μM) or Aicar (1 mM) for 1 h. The phosphorylation of AMPK was detected by Western blot assay. The values are presented as the means ± SDs (*n* = 3). * *p* < 0.05 and *** *p* < 0.001, compared to the negative control.

To investigate the underlying mechanism by which compounds **3** and **6** stimulate 2-NBDG uptake into cells, the effects of these compounds on the AMPK pathway were evaluated using differentiated mouse C2C12 skeletal myoblasts. As shown in Figure 5, phosphorylated AMPK was upregulated by treatment with 10 and 20 µM compounds **3** and **6** in differentiated C2C12 myoblasts. This result indicated that compounds **3** and **6** dose-dependently enhanced the phosphorylation of AMPK.

AMPK is a phylogenetically conserved fuel-sensing enzyme present in all mammalian cells that play an important role in the regulation of cellular energy homeostasis. It is activated in human skeletal muscles by events that increase the AMP/ATP ratio during exercise. Activated AMPK promotes glucose uptake, fatty acid oxidation, mitochondrial biogenesis, and insulin sensitivity, which is effective in preventing metabolic diseases such as obesity and diabetes [31]. The predominantly sedentary lifestyle of modern people that are lacking in physical activity ultimately leads to a decrease in activated AMPK in the body [9]. Thus, AMPK activators are emerging as important drug targets for metabolic diseases in modern society. Although many synthetic compounds have been developed against metabolic diseases, various adverse effects have also been reported [32]. Thus, plant-derived natural products have been studied as potential candidates for the management of these diseases [33]. Berberine, a natural isoquinoline alkaloid found in several medicinal plants, improves glucose metabolism by inducing AMPK activation and increasing glucose uptake in muscles and adipocytes [34]. Tetrahydrofuran lignans from *Myristica fragrans* (nutmeg) stimulate AMPK activation in differentiated C2C12 cells, and their mixtures exhibited a preventive effect on weight gain in a diet-induced animal model [35]. Hypoglycemic triterpenes from *Gynostemma pentaphyllum* significantly increased glucose uptake and GLUT4 translocation via activation of the AMPK/ACC signaling pathway [36]. Furthermore, selective activation of AMPK*α* by baicalein, a major flavonoid in *Scutellaria baicalensis*, ameliorated metabolic disorders in diet-induced mice [37]. Many plant-derived natural products have been demonstrated to be potential sources of antidiabetic drugs acting through AMPK activation. Although certain classes of natural products, including alkaloids, lignans, triterpenoids, and flavonoids, have been revealed as AMPK activators, this study suggested that macrocyclic glycosides (such as compound **3**) and biphenyl glycosides (such as compound **6**) are new classes of natural products candidates for use as T2DM therapeutics.

## 3. Materials and Methods

### 3.1. General Experimental Procedures

Optical rotations were measured on a JASCO P-2000 polarimeter using a 1-cm microcell. UV spectra were obtained on a SpectraMax M5 (Molecular Devices, Sunnyvale, CA, USA). Mass spectrometric data were acquired using a Waters Xevo G2 QTOF mass spectrometer (Waters MS Technologies, Manchester, UK), which was equipped with an electrospray interface (ESI) for high-resolution–electrospray ionization–mass spectrometry (HRESIMS). Nuclear magnetic resonance (NMR) spectra were obtained using Bruker AVANCE 500 MHz and 800 MHz NMR spectrometers with tetramethylsilane (TMS) as the internal standard, and chemical shifts are expressed as *δ* values. Infrared (IR) spectra were obtained using a JASCO FT/IR-4200 spectrometer. Thin-layer chromatography (TLC) analyses were performed on silica gel 60 F_254_ (Merck) and RP-18 F_254S_ (Merck) plates. Compounds were visualized by dipping the plates into 20% (*v*/*v*) H_2_SO_4_ reagent (Aldrich) and then heating at 110 °C for 5–10 min. Column chromatography (CC) was performed with reversed-phase (RP) silica gel (YMC Co., ODS-A 12 nm S-150 μm), Diaion HP-20 (Mitsubishi), and Sephadex LH-20 (Amersham Pharmacia Biotech). Medium-pressure liquid chromatography (MPLC) was performed using an MPLC-Reveleris system (Grace, IL, USA) with a Reveleris flash cartridge column (C18, 120 g). Semipreparative high-performance liquid chromatography (HPLC) was performed using a Gilson 321 pump, Gilson UV/VIS-151 detector (Gilson Inc., Middleton, WI, USA), and YMC-Triart C18 column (250 × 10 mm i.d., 5 μm, YMC Co., Ltd., Kyoto, Japan). All solvents were purchased from Daejung Chemicals (Daejung Chemicals & Metals Co., Ltd., Siheung, Korea). The authentic d- and L-glucose references were purchased from Sigma-Aldrich (St. Louis, MO, USA), and those of d-allose and L-allose were purchased from Alfa Aesar (Ward Hill, MA, USA) and Carbosnyth (Compton, Berkshire, UK), respectively. L-Cysteine methyl ester hydrochloride and *o*-tolyl isothiocyanate were obtained from Tokyo Chemical Industry Co., Ltd. (Tokyo, Japan).

### 3.2. Plant Material

The leaves of *Heliciopsis terminalis* (Proteaceae) were collected in Phu Luong district, Thai Nguyen province (GPS 21°52′47.5″ N 105°44′35.7″ E), Vietnam, in January 2018. The samples were identified based on their morphological characteristics by one of the authors (H.T.T.P). A voucher specimen was deposited in the Medicinal Herbarium of Hanoi University of Pharmacy with accession number HNIP.18510/15.

### 3.3. LC-MS/MS Analysis and Molecular Networking

The 70% MeOH extract, partitions, and fractions were analyzed by LC−MS/MS on a Waters Acquity UPLC BEH C18 (100 mm × 2.1 mm, 1.7 μm) column, of which the temperature was maintained at 40 °C. Solvent mixtures of H_2_O (A) and MeCN (B) were eluted at a flow rate of 0.3 mL/min with a linear gradient of 10–90% B (0–20 min) for the total extract and partitions and a linear gradient of 3–50% B (0–20 min) for the fractions. Samples were prepared at 2 mg/mL for the total extract and 1 mg/mL for the partitions and subfractions. One microliter of each sample was injected and analyzed in data-dependent acquisition (DDA) mode. A full MS survey scan was performed for 360 ms in the range of 50−1500 Da, and the three most intense ions were further scanned to acquire MS/MS fragmentation spectra. The gradient of collision energy was set to 20–80 V. MS/MS data were converted to mzXML format with MS-Convert35 and then uploaded onto the GNPS web platform (https://gnps.ucsd.edu (accessed on 13 September 2018)) for molecular networking. Parameters for molecular network generation were set as follows: precursor mass tolerance *m*/*z* of 0.02 Da, MS/MS fragment ion tolerance *m*/*z* of 0.02 Da, minimum cosine score of 0.7, minimum matched fragment ions of 4, minimum cluster size of 1, and network TopK of 10. Spectral library matching was performed with the same minimum cosine score and matched fragment ion number filtering parameter. The generated molecular network was visualized using Cytoscape 3.7.1. The MS/MS molecular network is accessible at the GNPS website with the following link: https://gnps.ucsd.edu/ProteoSAFe/status.jsp?task=fb3b5af8c1a044d598820f7ffdccb145 (accessed on 13 September 2018).

### 3.4. Extraction and Isolation

The dried leaves of *H. terminalis* (1.97 kg) were extracted with 10 L of 70% MeOH with ultrasonication at room temperature (90 min × 3 times), and the solvent was evaporated *in vacuo* at 40 °C. The 70% MeOH extract (98.6 g) was suspended in H_2_O and partitioned with *n*-hexane (1.41 g), EtOAc (6.7 g), *n*-BuOH (12.9 g), and water-soluble extract. The EtOAc-soluble fraction (6.7 g) was subjected to silica gel MPLC eluted with *n*-hexane/EtOAc/MeOH mixtures (1:0:0–0:0:1) to give 16 fractions (A–P). Fr. E (76.8 mg) was chromatographed over an RP-MPLC using a MeOH/H_2_O gradient solvent system (25:75 to 100% MeOH) to yield compound **10** (7.1 mg). Fr. N (370.2 mg) was separated by RP-MPLC with MeOH/H_2_O mixtures (10:90 to 100% MeOH) to give compound **8** (11.6 mg). The *n*-BuOH-soluble fraction (12.9 g) was chromatographed by Diaion HP-20 CC (*Φ* 5.0 × 30 cm) with a MeOH/H_2_O gradient (0:1 to 100% MeOH) as the mobile phase to afford 5 fractions (B.A–B.E). Fr. B.C (1.98 g) was separated by RP-MPLC using MeOH/H_2_O mixtures (1:19–3:2) to afford 6 subfractions (B.C1–B.C6). Subfraction B.C2 (700.0 mg) was separated by Sephadex LH-20 with MeOH to give five fractions (B.C2-1-B.C2-5). Compound **9** (15.7 mg) was obtained from fr. B.C2-2 (144.0 mg) by silica gel CC using EtOAc/MeOH/H_2_O mixtures (1:0:0–8:1.8:0.2). Compounds **6** (5.0 mg) and **7** (12.7 mg) were isolated by semipreparative HPLC (YMC Triart C18, 10 × 250 mm, 7%–20% MeCN in H_2_O (0.1% formic acid)) from fr. B.C2-4. Compound **5** (10.8 mg) was purified by Sephadex LH-20 with MeOH from fr. B.C4 (306.0 mg). Fraction B.D (1.67 g) was separated by silica gel MPLC using *n*-hexane/EtOAc/MeOH mixtures (7:3:0–0:0:1) to yield 11 subfractions (B.D1–B.D11). Fr. B.D8 (50.1 mg) was further purified by semipreparative HPLC (YMC Triart C18, 10 × 250 mm, 20% MeCN in H_2_O (0.1% formic acid)) to afford compounds **1** (4.6 mg), **2** (4.3 mg), **3** (3.9 mg) and **4** (4.6 mg).

### 3.5. Spectroscopic and Physical Characteristics of Compounds

*Heliciopside A (**1**)*: White amorphous powder; ${\left[a\right]}_{D}^{20}$ + 15 (*c* 0.5, Pyridine); UV (MeOH) *λ*_max_ (log *ε*) 201 (4.79), 244 (4.36) nm; IR (KBr) *ν*_max_ 3393, 1710, 1605, 1509, 1290, 1087, 1046 cm^−1^; ^1^H and ^13^C NMR data, see Table 1; HRESIMS *m*/*z* 563.1399 [M − H]^−^ (calcd for C_26_H_27_O_14_, 563.1401).

*Heliciopside B (**2**)*: White amorphous powder; ${\left[a\right]}_{D}^{20}$ + 14 (*c* 0.5, Pyridine); UV (MeOH) *λ*_max_ (log *ε*) 201 (4.76), 243 (4.33) nm; IR (KBr) *ν*_max_ 3277, 1704, 1606, 1509 cm^−1^; ^1^H and ^13^C NMR data, see Table 1; HRESIMS *m*/*z* 563.1403 [M − H]^−^ (calculated for C_26_H_27_O_14_, 563.1401).

*Heliciopside C (**3**)*: White amorphous powder; ${\left[a\right]}_{D}^{20}$ + 34 (*c* 0.5, Pyridine); UV (MeOH) *λ*_max_ (log *ε*) 204 (4.39), 251 (4.02) nm; IR (KBr) *ν*_max_ 3384, 1712, 1595, 1509 cm^−1^; ^1^H and ^13^C NMR data, see Table 1; HRESIMS *m*/*z* 623.1608 [M − H]^−^ (calculated for C_28_H_31_O_16_, 623.1612).

*Heliciopside D (**5**)*: White amorphous powder; ${\left[a\right]}_{D}^{20}$ − 38 (*c* 0.5, MeOH); UV (MeOH) *λ*_max_ (log *ε*) 203 (4.61), 251 (4.12) nm; IR (KBr) *ν*_max_ 3359, 1697, 1606, 1508 cm^−1^; ^1^H and ^13^C NMR data, see Table 1; HRESIMS *m*/*z* 627.1907 [M − H]^−^ (calculated for C_28_H_35_O_16_, 627.1925).

*Heliciopside E (**6**)*: Brown amorphous solid; ${\left[a\right]}_{D}^{20}$ + 14 (*c* 0.5, MeOH); UV (MeOH) *λ*_max_ (log *ε*) 206 (3.43), 289 (2.74) nm; IR (KBr); *ν*_max_ 3392, 1711, 1668, 1623, 1511 cm^−1^; ^1^H and ^13^C NMR data, see Table 2; HRESIMS *m*/*z* 897.2102 [M − H]^−^ (calculated for C_42_H_41_O_22_, 897.2089).

### 3.6. Determination of the Absolute Configurations of the Sugars in Compounds **1**–**3**, **5** and **6**

Compounds **1**–**3**, **5**, and **6** (each 0.5 mg) were hydrolyzed with 1 M HCl (1.0 mL) at 90 °C for 2 h to yield aglycone and sugar. The reaction mixture was partitioned with EtOAc (1.0 mL) to give an aqueous fraction containing the sugar. After the aqueous fractions were dried with an N_2_ condenser, L-cysteine methyl ester hydrochloride (0.5 mg) in anhydrous pyridine was added and heated at 60 °C for 1 h. Each mixture was added to *o*-tolylisothiocyanate (1 μL) and continuously heated at 60 °C for 1 h. The final reaction mixture was directly analyzed by RP-HPLC using a Thermo Scientific Dionex Ultimate 3000 UHPLC system. The column used was a 250 mm × 4.6 mm i.d., 5 μm, Kinetex 5 μm C18 column; the mobile phase was MeCN/H_2_O containing 0.1% formic acid (25:75, *v*/*v*); detection was performed by UV at 250 nm, the flow rate was 0.8 mL/min, and the column temperature was 35 °C. The identities of the sugar derivatives were determined by comparing the retention times (*t*_R_s) with those of the derivatives of commercial d-glucose (*t*_R_ = 11.45 min), L-glucose (*t*_R_ = 10.60 min), d-allose (*t*_R_ = 11.69 min), and L-allose (*t*_R_ = 8.52 min) (Supporting Information). Thus, the glucose and allose moieties in compounds **1**–**3**, **5** and **6** were determined to be the d-configuration.

### 3.7. Differentiation of 3T3-L1 Adipocytes

3T3-L1 preadipocyte cells were incubated in Dulbecco’s modified Eagle’s medium (DMEM) (HyClone, Logan, UT, USA) supplemented with 10% calf serum, 100 μg/mL streptomycin, and 100 U/mL penicillin (Gibco, Grand Island, NY, USA) at 37 °C in 5% CO_2_. For differentiation, the culture medium was changed to DMEM with 10% fetal bovine serum (FBS) (HyClone), 1 μM dexamethasone (Sigma), 520 μM 3-isobutyl-1-methylxanthine (Sigma), and 1 μg/mL insulin (Roche, Mannheim, Germany). After 2 days of incubation, the cells were replenished with fresh DMEM containing 10% FBS, 1 μg/mL insulin, 100 μg/mL streptomycin, and 100 U/mL penicillin. The medium was replaced every 1.5−2 days until adipogenesis induction.

### 3.8. Cell Viability Assay

The 3-(4,5-dimethyl-2-thiazolyl)-2,5-diphenyl-2H-tetrazolium bromide (MTT) method was applied to evaluate cell viability. Briefly, 3T3-L1 adipocytes were seeded on 96-well plates and grown using DMEM supplemented with 10% FBS. After one day of incubation, the cells were treated with the test compounds that were dissolved in a serum-free medium for a day. Then, 20 μL of 2 mg/mL MTT solution (Sigma) was added to each well and incubated for 4 h in the dark. After removing the supernatant, formazan was dissolved in DMSO, and the absorbance was measured at 550 nm using a microplate reader (VersaMax, PA, USA).

### 3.9. Measurement of Glucose Uptake Using the 2-NBDG Probe

To measure glucose uptake level, 2-NBDG (Invitrogen, Eugene, OR, USA), a fluorescent derivative of glucose, was used. The 3T3-L1 adipocytes were grown on 96-well plates using a medium without glucose with 10% FBS, as previously described [21]. After 24 h of incubation, the cells were treated with insulin as a positive control and the test samples in the presence or absence of 2-NBDG. Then, the cultures were incubated for 1 h, and the cells were washed with cold phosphate-buffered saline (PBS). The fluorescence signal intensity was measured at 450 nm excitation and 535 nm emission wavelengths using a fluorescence microplate reader (Spectra Max Gemini XPS, Molecular Devices, San Jose, CA, USA) for quantifying 2-NBDG fluorescence. Additionally, the 3T3-L1 adipocytes were plated on sterilized glass coverslips in glucose-free medium containing 10% FBS for 24 h, and fluorescence images were taken using a fluorescence microscope (Olympus ix70, Olympus Corporation, Tokyo, Japan) to analyze the transport of 2-NBDG into the cells.

### 3.10. Differentiation of C2C12 Myoblasts

C2C12 myoblasts were incubated with DMEM containing 10% FBS and 1% penicillin 10,000 U/mL, and 10,000 μg/mL streptomycin. C2C12 myoblasts were cultured for 2 days post-confluence for differentiation. The medium was replenished with fresh DMEM medium with 2% horse serum (Gibco, NY, USA) every 2 days until the myotube formed.

### 3.11. Detection of p-AMPKα Thr^172^ by Western blotting

The differentiated C2C12 cells were washed with PBS and lysed using lysis buffer [50 mM Tris-HCl (pH = 7.6), 50 mM NaF, 1 mM EDTA, 120 mM NaCl, 0.5% NP-40] containing protease and phosphatase inhibitors (Roche), and were centrifuged for 12 min at 12,000 rpm at 4 °C. The protein was taken in the supernatant of the sample, and the protein concentration was measured with a protein assay kit (Bio-Rad Laboratories, CA, USA). The extract volumes were adjusted with the same lysis buffer, and each of them was loaded for SDS–PAGE and transferred to PVDF membranes (PVDF 0.45 μm, Immobilon-P, Millipore, Billerica, MA, USA). The blocking process was performed with 5% (*w*/*v*) nonfat milk for 1 h with shaking at room temperature. After this, the membranes were incubated with primary antibodies (*p*-AMPK*α* Thr^172^, AMPK*α*, mouse monoclonal actin) overnight at 4 °C. The membranes were incubated continually with secondary antibodies at room temperature for 1 h, and the band signals were detected with the enhanced chemiluminescence (ECL) solution method. The results were measured with a LAS 4000 image processing system (Fuji Film, Tokyo, Japan).

### 3.12. Statistical Analysis

Data were processed through one-way analysis of variance (ANOVA) to evaluate variances between each group, followed by Tukey’s or Duncan’s post hoc test using GraphPad Prism 5 (GraphPad Software, Inc., San Diego, CA, USA). The results are expressed as means ± SD of three independent experiments. Statistical significance (*p*-value < 0.05) was considered (* *p* < 0.05, ** *p* < 0.01, and *** *p* < 0.001).

## 4. Conclusions

We investigated the chemical profiles of *H. terminalis* leaves by applying LC-MS/MS molecular networking and 10 phenolic constituents, including three new macrocyclic glycosides (**1**–**3**), a new phenolic glycoside (**5**), a new biphenyl glycoside (**6**), and five known compounds (**4**, **7**–**10**), were isolated from the leaves of *H. terminalis*. Many plants belonging to the family Proteaceae have been reported to contain phenolic glycosides, such as arbutin derivatives [38,39,40]. However, in this paper, a macrocyclic glycoside and a biphenyl glycoside were identified from the *H. terminalis* (Proteaceae) for the first time. Macrocyclic glycosides are secondary metabolites rarely found in nature, accounting for only approximately ten of the related compounds reported to date, and even these have been mostly isolated from the genera *Clematis* and *Berchemia* [22,28,41,42,43]. Since these metabolites have chiral cavities with both hydrophilic and hydrophobic functionalities, such as glycophane, various biological activities and applications could be expected [44]. In this study, all isolates (**1**–**10**), including macrocyclic glycosides **1**–**4**, were evaluated for their stimulatory effects on the uptake of 2-NBDG in 3T3-L1 adipocytes. Notably, compounds **3**, **4**, and **6**–**9** exhibited effects on glucose uptake without cytotoxicity. Among macrocyclic glycosides **1**–**4**, compounds **3** and **4** exhibited significant moderate glucose uptake effects, respectively, whereas **1** and **2** had no considerable effects. From these results, we inferred that methoxy groups on the phenolic acid moiety might increase the stimulation of glucose uptake. Further investigation of compounds **3** and **6**, which possessed the most potent stimulatory effects on glucose uptake, revealed that these compounds act by stimulating AMPK-mediated glucose uptake. Our results suggest for the first time that the phenolic constituents in *H. terminalis* have the potential as anti-diabetic agents.

## Figures and Tables

**Table 1:** ^1^H- and ^13^C-NMR spectroscopic data of compounds **1**–**3** and **5** in DMSO-*d*_6_.

No.	1 ^a^		2 ^a^		3 ^a^		5 ^b^	
	*δ*_C_	*δ*_H_ (*J* in Hz)	*δ*_C_	*δ*_H_ (*J* in Hz)	*δ*_C_	*δ*_H_ (*J* in Hz)	*δ*_C_	*δ*_H_ (*J* in Hz)
**1**	122.4	-	122.6	-	124.6	-	153.6	-
**2**	131.3	8.02, d (8.8)	131.2	7.99, d (8.8)	107.0	7.09, d (1.6)	94.3	6.32, s
**3**	115.8	7.19, d (8.8)	115.8	7.19, d (8.8)	153.1	-	153.1	-
**4**	160.6	-	160.4	-	137.7	-	132.6	-
**5**	115.8	7.19, d (8.8)	115.8	7.19, d (8.8)	152.0	-	153.1	-
**6**	131.3	8.02, d (8.8)	131.2	7.99, d (8.8)	106.7	7.52, d (1.6)	94.3	6.32, s
**7**	165.1	-	165.0	-	164.9	-		-
	d-allo		d-glc		d-glc		d-glc	
**1′**	96.8	5.39, d (8.0)	98.4	5.22, d (7.2)	100.9	5.36, d (7.2)	100.2	4.98, d (8.0)
**2′**	69.9	3.56, dd (8.0, 2.4)	72.9	3.34, m	73.9	3.30, overlap	73.1	3.26, overlap
**3′**	71.5	3.99, dd (2.4, 2.4)	76.7	3.38, m	76.7	3.30, overlap	76.3	3.32, overlap
**4′**	68.3	3.45, dd (9.6, 2.4)	70.7	3.17, m	71.1	3.12, dd (9.6, 8.8)	70.0	3.26, overlap
**5′**	71.2	4.29, ddd (10.4, 9.6, 2.4)	73.5	3.97, m	73.8	3.49, dd (10.4, 2.4)	73.7	3.80, m
**6a′** **6b′**	65.4	4.37, dd (11.2, 1.6) 4.11, dd (11.2, 10.4)	65.0	4.40, dd (11.2, 2.4) 4.07, br d (11.2)	64.9	4.40, br d (11.2) 3.89, dd (11.2, 10.4)	64.2	4.58, br d (11.5) 4.23, dd (11.5, 7.0)
**1″**	-	-	122.5	-	122.5	-	122.9	-
**2″**	-	-	131.2	7.98, d (8.8)	130.7	7.45, d (8.8)	131.1	7.86, d (8.5)
**3″**	-	-	115.8	7.18, d (8.8)	115.9	6.98, d (8.8)	155.9	7.09, d (8.5)
**4″**	-	-	160.7	-	160.7	-	161.4	-
**5″**	-	-	115.8	7.18, d (8.8)	115.9	6.98, d (8.8)	155.9	7.09, d (8.5)
**6″**	-	-	131.2	7.98, d (8.8)	130.7	7.45, d (8.8)	131.1	7.86, d (8.5)
**7″**	-	-	165.1	-	164.87	-	165.3	-
			d-allo		d-allo		d-allo	
**1** **‴**	-	-	96.8	5.37, d (8.0)	97.6	5.17, d (8.0)	98.1	5.23, d (7.5)
**2** **‴**	-	-	69.9	3.54, m	70.0	3.53, dd (8.0, 2.4)	70.2	3.46, overlap
**3** **‴**	-	-	71.5	3.97, m	71.5	3.99, m	71.5	3.94, m
**4** **‴**	-	-	68.2	3.43, m	68.3	3.45, m	66.9	3.46, overlap
**5** **‴**	-	-	71.2	4.26, ddd (10.4, 10.4, 1.6)	71.6	4.28, ddd (10.4, 10.4, 1.6)	74.7	3.73, m
**6a‴** **6b‴**	-	-	65.4	4.36, dd (11.2, 1.6) 4.09, br d (11.2)	64.8	4.50, dd (11.2, 11.2) 4.40, br d (11.2)	60.8	3.68, m 3.46, overlap
**3-OMe**	-	-	-	-	56.0	3.61, s	55.8	3.65, s
**4-OMe**	-	-	-	-	-	-	60.2	3.57, s
**5-OMe**	-	-	-	-	56.5	3.98, s	55.8	3.65, s

^a 1^H- and ^13^C-NMR spectra were acquired at 800 and 200 MHz, respectively. ^b 1^H- and ^13^C-NMR spectra were acquired at 500 and 125 MHz, respectively.

**Table 2:** ^1^H (800 MHz) and ^13^C (200 MHz) NMR spectroscopic data of compound **6** in CD_3_OD.

No.	*δ*_C_	*δ*_H_ (*J* in Hz)	No.	*δ*_C_	*δ*_H_ (*J* in Hz)	No.	*δ*_C_	*δ*_H_ (*J* in Hz)
**1**	148.8		**1′**	103.3	4.63, d (7.7)	**1″**	70.4	-
**2**	106.7	6.66, s	**2′**	74.8	3.27, m	**2″**,**6″**	151.2	6.89, m
**3**	141.5		**3′**	77.7	3.33, overlap	**3″**,**5″**	128.7	6.22, m
**4**	145.9		**4′**	71.4	3.33, overlap	**4″**	187.2	-
**5**	119.1	6.62, s	**5′**	75.3	3.49, m	**7″**	148.1	6.71, d (16.2)
**6**	121.4		**6′a** **6′b**	64.8	4.44, dd (11.2, 2.4) 4.29, dd (11.2, 6.4)	**8″**	122.9	6.30, d (16.2)
						**9″**	167.4	-

## Data Availability

Data is contained within the article and Supplementary Material.

## Supplementary Materials

The following are available online at https://www.mdpi.com/article/10.3390/ph15111315/s1. Figure S1: MS chromatogram of total extract, *n*-hexane, EtOAc, *n*-BuOH and water fractions of *H. terminalis*. Figures S2–S46: HRESIMS, 1D and 2D NMR, IR, and UV spectra of compounds **1**–**3**, **5** and **6**. Figure S47: Effects of compounds **1**–**10** on 2-NBDG uptake in 3T3-L1 adipocytes. The fluorescence and bright-field images were captured by the fluorescence microscopy method. Figure S48: Stimulation effects of compounds 3 and 6 at different concentrations (5, 10 and 20 μM) on 2-NBDG uptake using 3T3-L1 adipocytes. The fluorescence and bright-field images were captured by the fluorescence microscopy method. Figure S49: The cytotoxicity effects of compounds **1**–**10** at a concentration of 20 μM (A) in 3T3-L1 adipocytes. The cells were incubated with compounds for 24 h at 37 °C. Then, the MTT assay was performed as described in the experimental section. The results were calculated as the means ± SDs (*n* = 3), * *p* < 0.05, ** *p* < 0.01 and *** *p* < 0.001, compared to the negative control. Figure S50: The cytotoxicity effects of compound **10** at different concentrations (5, 10 and 20 μM) in 3T3-L1 adipocytes. Figure S51: The effects of compounds **3** and **6** on p-AMPK (Thr172) in C2C12 cells; original blot images. The expression of *p*-AMPK was firstly observed. The bounding antibodies were removed by using a Restore^TM^ Western blot stripping buffer (Thermo Sci.). The blot was continuously stripped and incubated with AMPK and *β*-actin antibodies. Blots from three independent experiments were shown (A–C). Sample names were from left as follows: Ctrl, Aicar (1 mM), compound **3** (10 μM), compound **3** (20 μM), compound **6** (10 μM), and compound **6** (20 μM). * *p* < 0.05, ** *p* < 0.01, and *** *p*< 0.001, compared to negative control. HPLC chromatogram of chiral derivatives from compounds **1**–**3**, **5** and **6** and authentic samples.
