# Supplementary material for: Heliciopsides A−E, Unusual Macrocyclic and Phenolic Glycosides from the Leaves of Heliciopsis terminalis and Their Stimulation of Glucose Uptake
Source: Pharmaceuticals (Basel). 2022 Oct 25;15(11):1315. doi: 10.3390/ph15111315 (PMC9695999; doi:10.3390/ph15111315)
Supplement: Supplementary file 1 [file pharmaceuticals-15-01315-s001.zip › pharmaceuticals-1940838-supplementary.pdf]

Supplementary Materials

Heliciopsides A−E, Unusual Macrocyclic and Phenolic Glycosides from the Leaves of *Heliciopsis terminalis* and Their Stimulation of Glucose Uptake

Byeol Ryu [1], Eun-Jin Park [1], Thi-Phuong Doan [1], Hyo-Moon Cho [1], Jin-Pyo An [1], Thi-Linh-Giang Pham [2], Ha Thanh Tung Pham [2], Won Keun Oh [1,*]

[1] Korea Bioactive Natural Material Bank, Research Institute of Pharmaceutical Sciences, College of Pharmacy, Seoul National University, Seoul 08826, Korea;
[2] Department of Botany, Hanoi University of Pharmacy, Hanoi 000084, Vietnam
* Correspondence: wkoh1@snu.ac.kr; Tel.: +82-2-880-7872

Table of Contents

Figure S1. MS chromatogram of total extract, *n*-hexane, EtOAc, *n*-BuOH and water fractions of *H. terminalis*. 5

Figure S2. HRESI(−)MS spectrum of compound **1** 6

Figure S3. ^{1}H NMR spectrum of compound **1** in DMSO-d_6 7

Figure S4. ^{13}C NMR spectrum of compound **1** in DMSO-d_6 7

Figure S5. ^{1}H−^{1}H COSY spectrum of compound **1** in DMSO-d_6 8

Figure S6. HSQC spectrum of compound **1** in DMSO-d_6 8

Figure S7. HMBC spectrum of compound **1** in DMSO-d_6 9

Figure S8. ROESY spectrum of compound **1** in DMSO-d_6 9

Figure S9. IR spectrum of compound **1** 10

Figure S10. UV spectrum of compound **1** 10

Figure S11. HRESI(−)MS spectrum of compound **2** 11

Figure S12. ^{1}H NMR spectrum of compound **2** in DMSO-d_6 12

Figure S13. ^{13}C NMR spectrum of compound **2** in DMSO-d_6 13

Figure S14. ^{1}H−^{1}H COSY spectrum of compound **2** in DMSO-d_6 13

Figure S15. HSQC spectrum of compound **2** in DMSO-d_6 13

Figure S16. HMBC spectrum of compound **2** in DMSO-d_6 14

Figure S17. ROESY spectrum of compound **2** in DMSO-d_6 15

Figure S18. IR spectrum of compound **2** 15

Figure S19. UV spectrum of compound **2** 16

Figure S20. HRESI(−)MS spectrum of compound **3** 17

Figure S21. ^{1}H NMR spectrum of compound **3** in DMSO-d_6 18

Figure S22. ^{13}C NMR spectrum of compound **3** in DMSO-d_6 18

Figure S23. ^{1}H−^{1}H COSY spectrum of compound **3** in DMSO-d_6 19

Figure S24. HSQC spectrum of compound **3** in DMSO-d_6 19

Figure S25. HMBC spectrum of compound **3** in DMSO-d_6 20

Figure S26. ROESY spectrum of compound **3** in DMSO-d_6 20

Figure S27. IR spectrum of compound **3** 21

Figure S28.UV spectrum of compound **3** 21

Figure S29. HRESI(−)MS spectrum of compound **5** 22

Figure S30. ^{1}H NMR spectrum of compound **5** in DMSO-d_6 23

Figure S31. ^{13}C NMR spectrum of compound **5** in DMSO-d_6 23

Figure S32. ^{1}H−^{1}H COSY spectrum of compound **5** in DMSO-d_6 24

Figure S33. HSQC spectrum of compound **5** in DMSO-d_6 24

Figure S34. HMBC spectrum of compound **5** in DMSO-d_6 25

Figure S35. ROESY spectrum of compound **5** in DMSO-d_6 25

Figure S36. IR spectrum of compound **5** 26

Figure S37. UV spectrum of compound **5** 26

Figure S38. HRESI(−)MS spectrum of compound **6** 27

Figure S39. ^{1}H NMR spectrum of compound **6** in CD_3OD 28

Figure S40. ^{13}C NMR spectrum of compound **6** in CD_3OD 28

Figure S41. ^{1}H−^{1}H COSY spectrum of compound **6** in CD_3OD 29

Figure S42. HSQC spectrum of compound **6** in CD_3OD 29

Figure S43. HMBC spectrum of compound **6** in CD_3OD 30

Figure S44. ROESY spectrum of compound **6** in CD_3OD 30

Figure S45. IR spectrum of compound **6** 31

Figure S46. UV spectrum of compound **6** 31

Figure S47. Effects of compounds **1-10** on 2-NBDG uptake in 3T3-L1 adipocytes. The fluorescence and bright-field images were captured by the fluorescence microscopy method. 32

Figure S48. Stimulation effects of compounds **3** and **6** at different concentrations (5, 10 and 20 μM) on 2-NBDG uptake using 3T3-L1 adipocytes. The fluorescence and bright-field images were captured by the fluorescence microscopy method. 32

Figure S49. The cytotoxicity effects of compounds **1−10** at a concentration of 20 μM (A) in 3T3-L1 adipocytes. The cells were incubated with compounds for 24 h at 37°C. Then, the MTT assay was performed as described in the experimental section. The results were calculated as the mean ± SD (n = 3), * $p < 0.05$, ** $p < 0.01$ and *** $p < 0.001$, compared to the negative control. 33

Figure S50. The cytotoxicity effects of compound **10** at different concentrations (5, 10 and 20 μM) in 3T3-L1 adipocytes. 33

Figure S51. The effects of compounds **3** and **6** on *p*-AMPK (Thr172) in C2C12 cells; original blot images. The expression of *p*-AMPK was firstly observed. The bounding antibodies were removed by using a Restore™ Western blot stripping buffer (Thermo Sci.). The blot was continuously stripped and incubated with AMPK and β-actin antibody. Blots from three independent experiments were shown (A-C). Sample names were from left as follows: Ctrl, Aicar (1 mM), compound **3** (10 μM), compound **3** (20 μM), compound **6** (10 μM), and compound **6** (20 μM). *p < 0.05, **p < 0.01, and ***p< 0.001, compared to negative control. 33

HPLC chromatogram of chiral derivatives from compounds 1−3, 5 and 6 and authentic samples 34

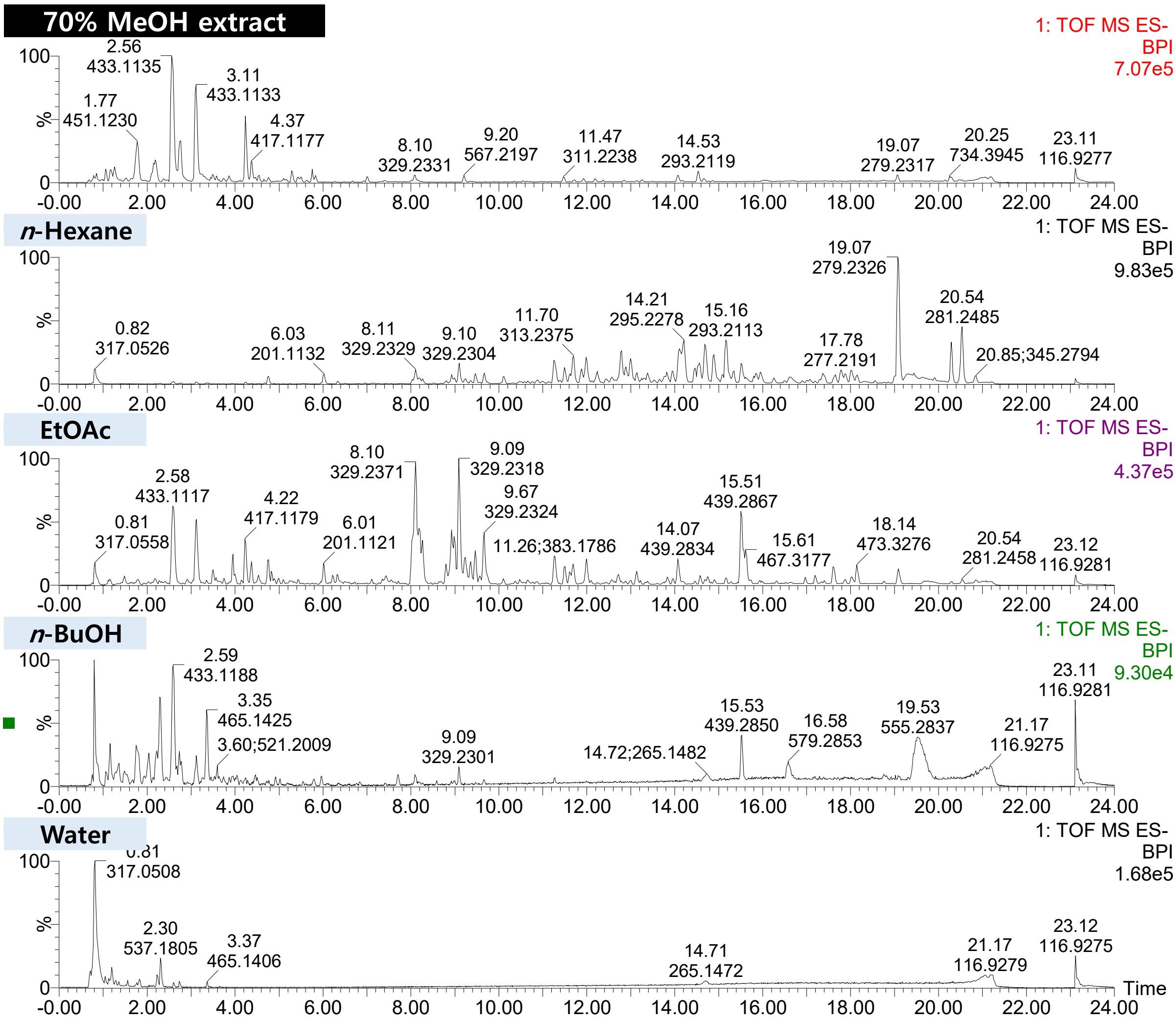

Figure S1. MS chromatogram of total extract, *n*-hexane, EtOAc, *n*-BuOH and water fractions of *H. terminalis*.

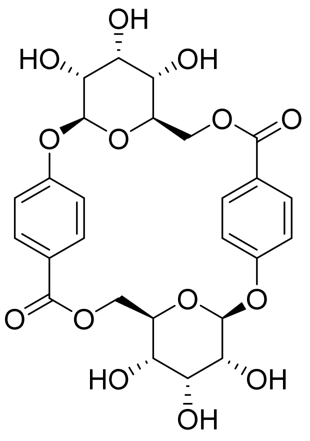

Structure of Compound **1**

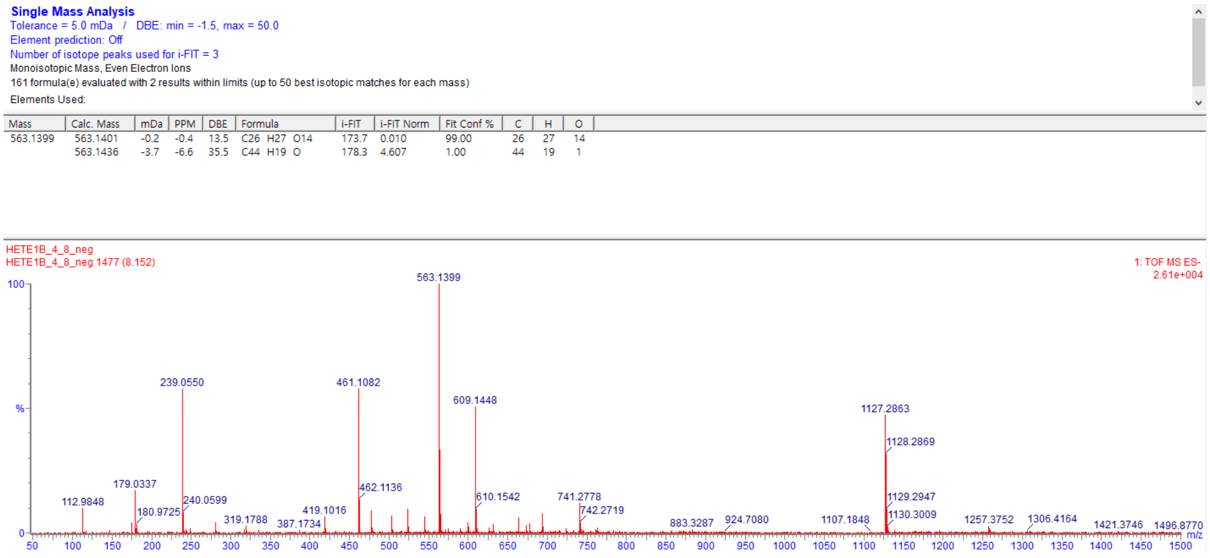

Mass	Calc. Mass	mDa	PPM	DBE	Formula	i-FIT	i-FIT Norm	Fit Conf %	C	H	O
563.1399	563.1401	-0.2	-0.4	13.5	C26 H27 O14	173.7	0.010	99.00	26	27	14
	563.1436	-3.7	-6.6	35.5	C44 H19 O	178.3	4.607	1.00	44	19	1

Figure S2. HRESI(−)MS spectrum of compound **1**

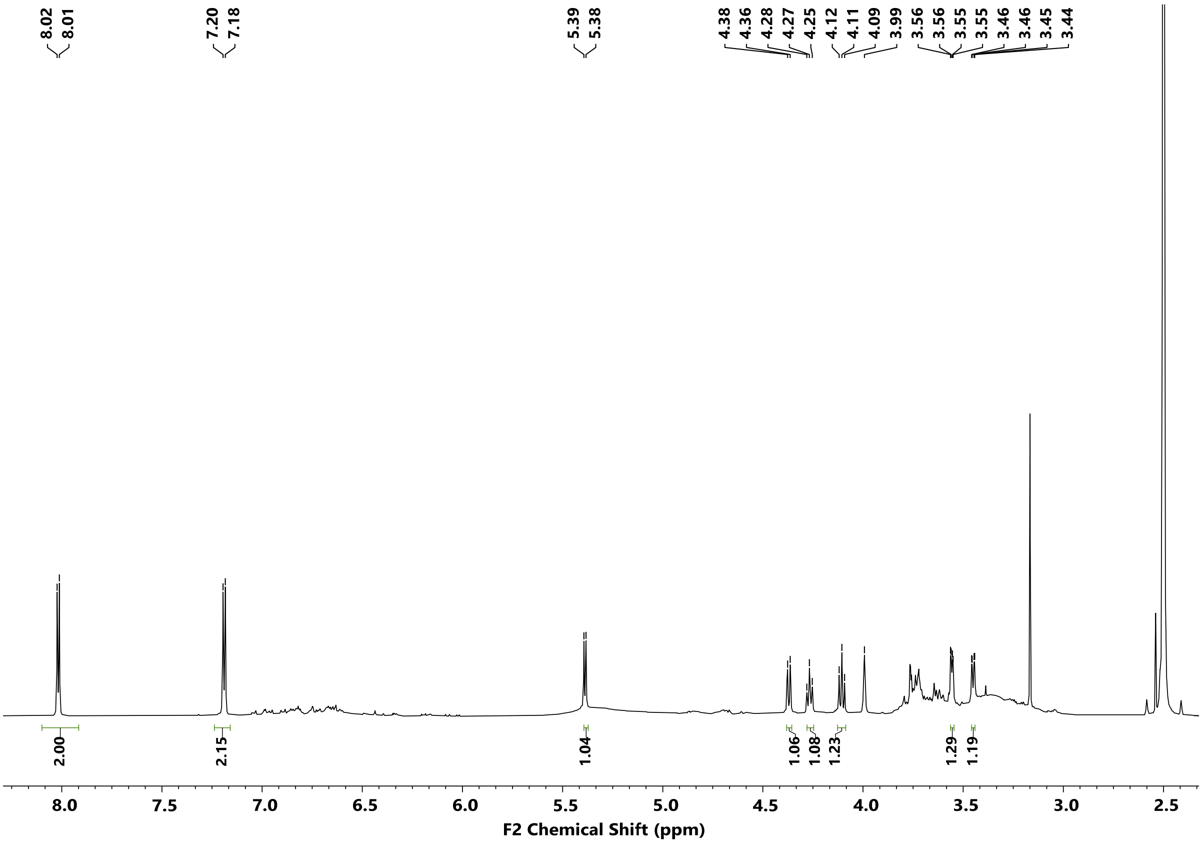

Figure S3. ^{1}H NMR spectrum of compound **1** in DMSO-d_6

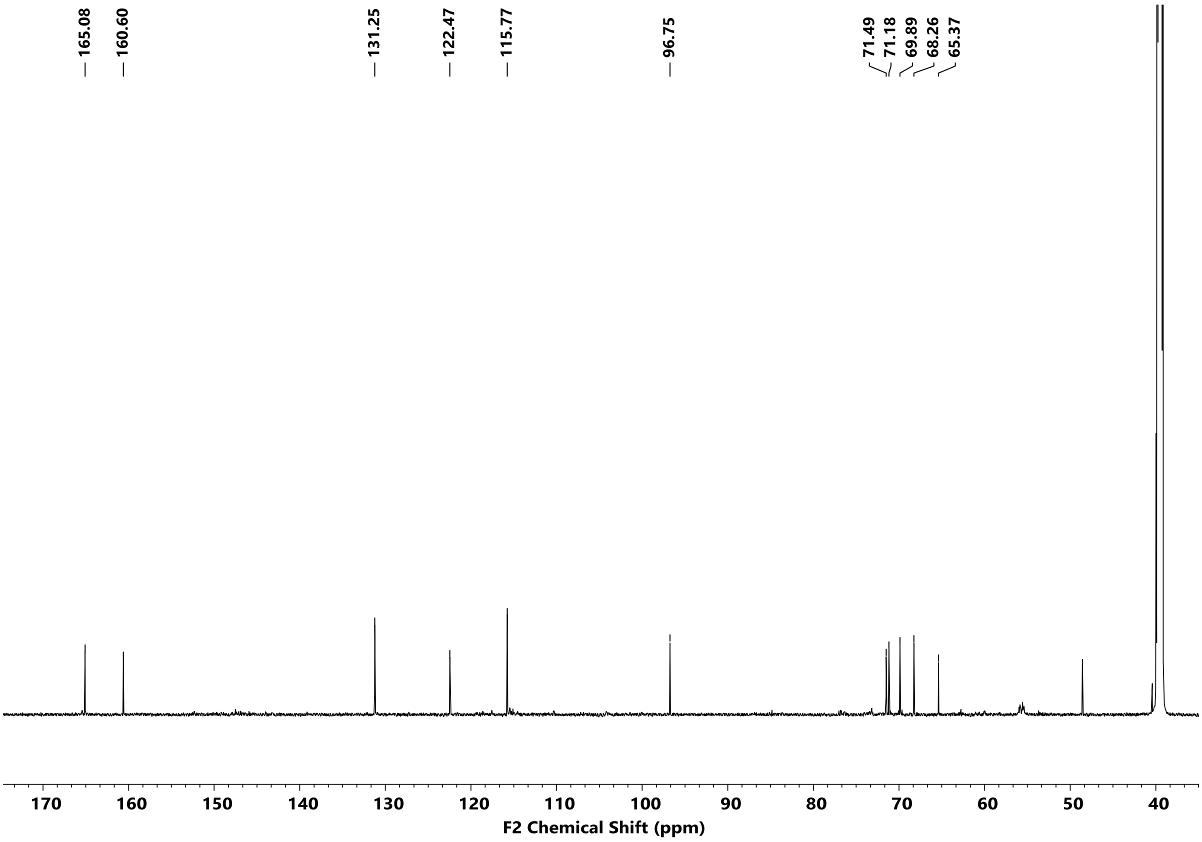

Figure S4. ^{13}C NMR spectrum of compound **1** in DMSO-d_6

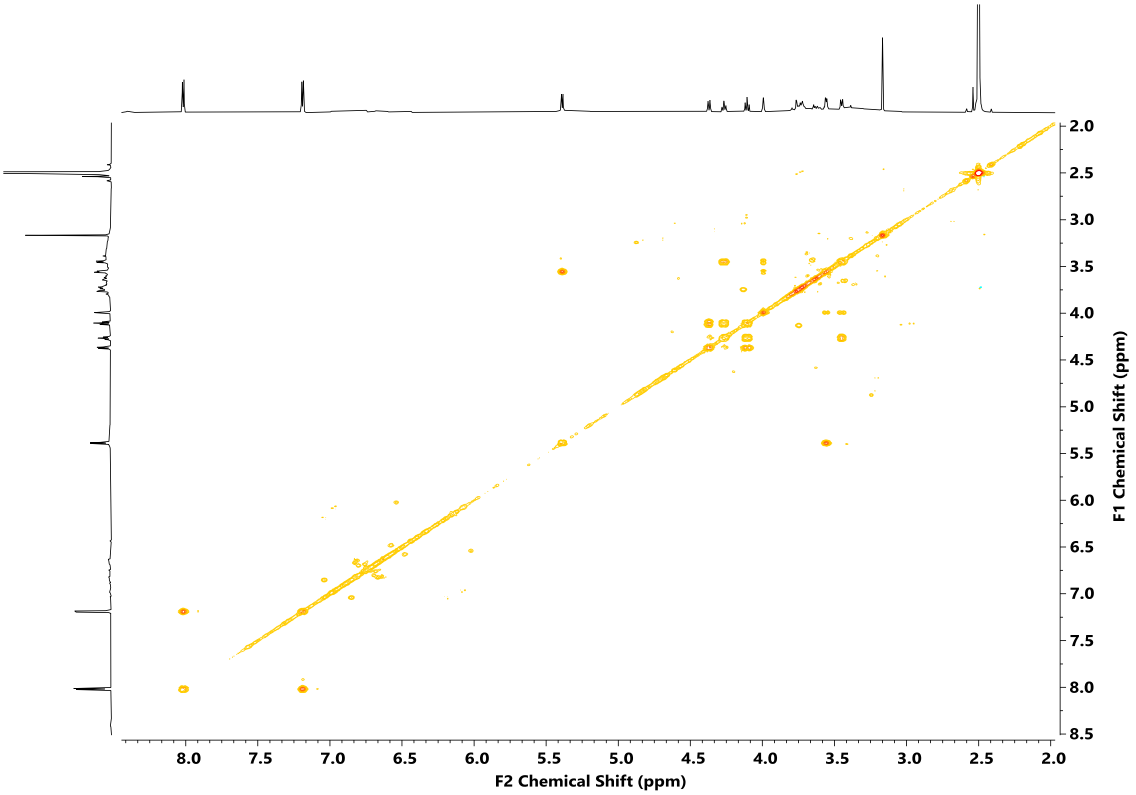

Figure S5. ^{1}H–^{1}H COSY spectrum of compound **1** in DMSO-*d*6

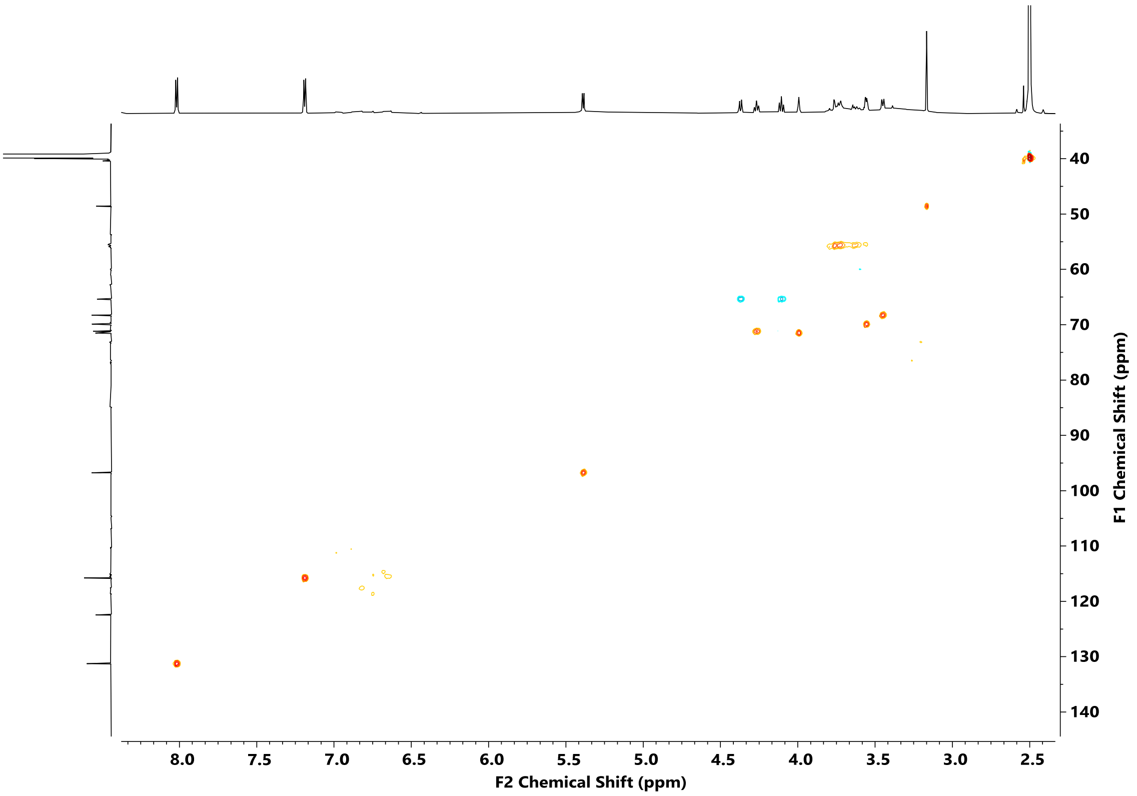

Figure S6. HSQC spectrum of compound **1** in DMSO-*d*6

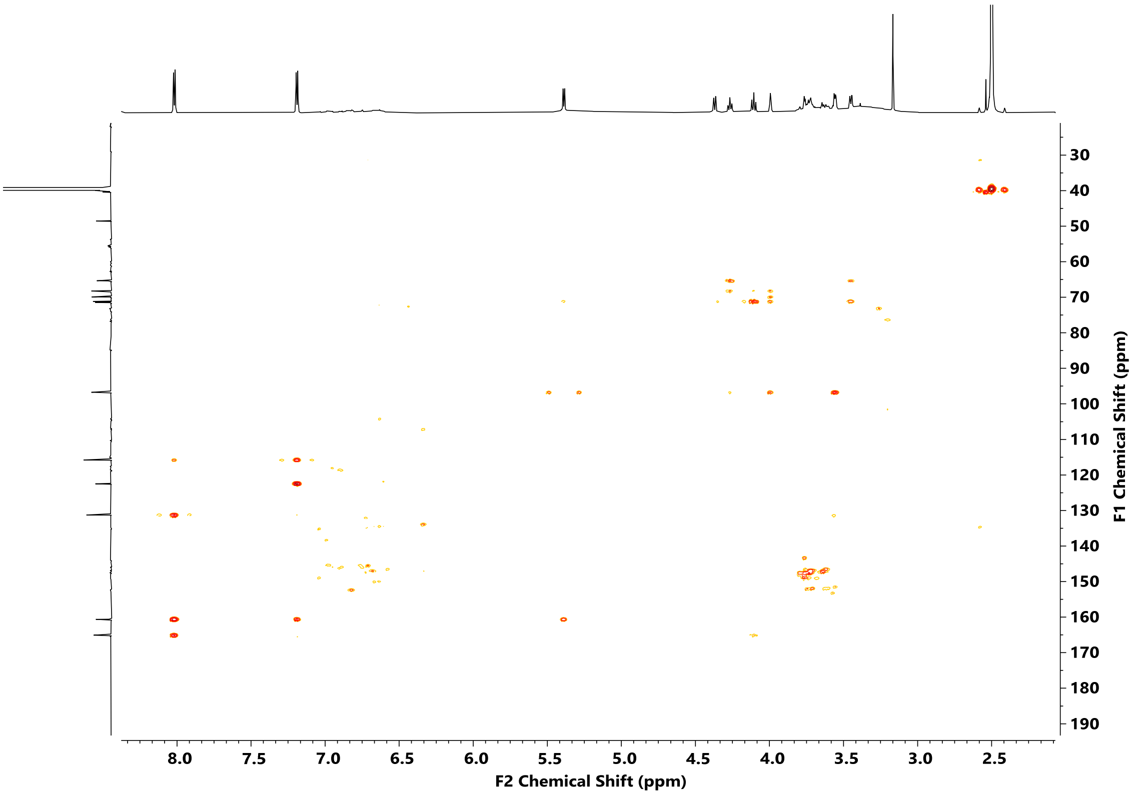

Figure S7. HMBC spectrum of compound **1** in DMSO-d_6

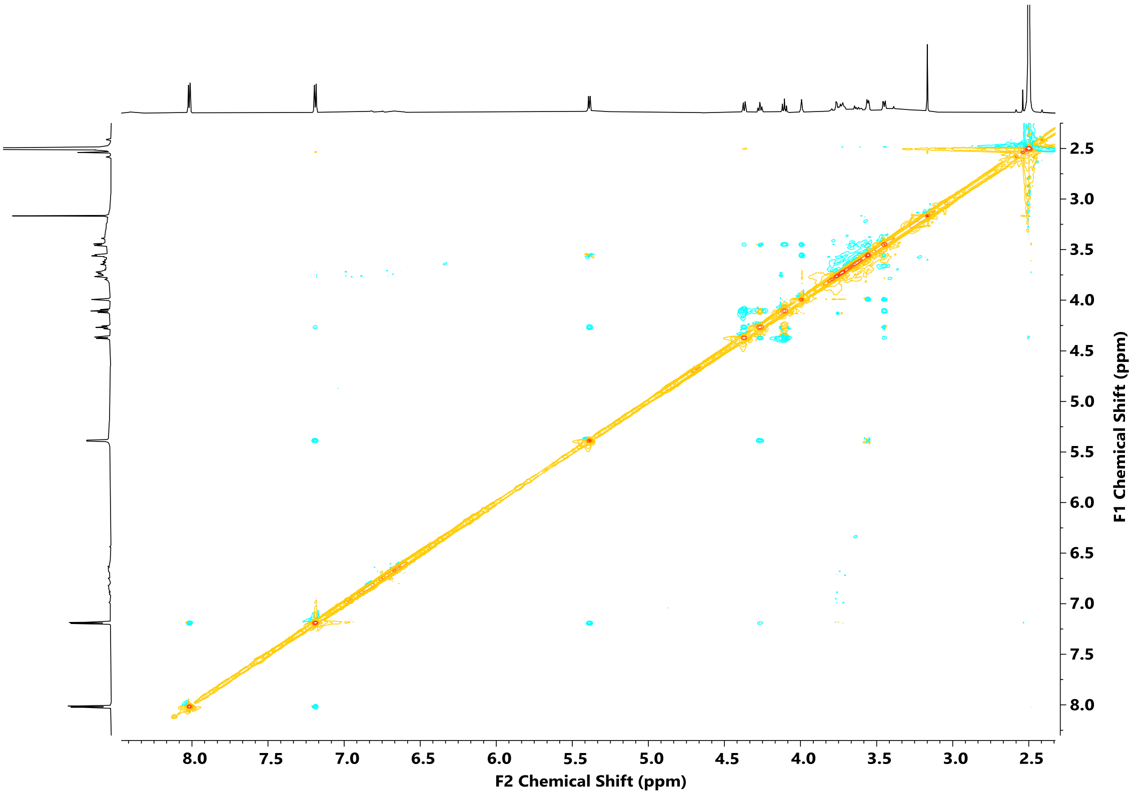

Figure S8. ROESY spectrum of compound **1** in DMSO-d_6

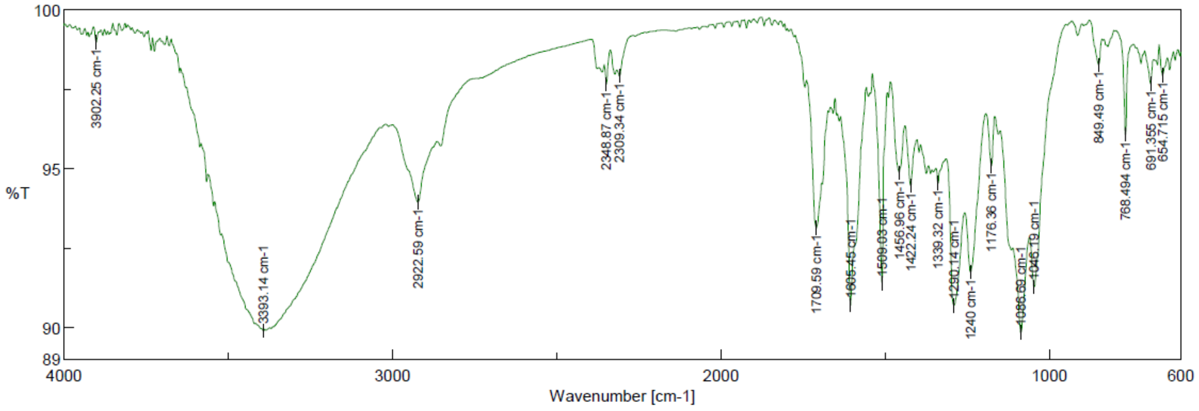

[Comment]
Sample Name 190729_HETE1E-S7
Comment
User
Division
Company 공동기기실

[Data Information]
Creation Date 2019-09-26 오후 4:30
Data array type Linear data array
Horizontal Wavenumber [cm-1]
Vertical %T
Start 599.753 cm-1
End 4000.6 cm-1
Data pitch 0.964233 cm-1
Data points 3528

[Measurement Information]
Model Name FT/IR-4200typeA
Serial Number B038361018

Light Source Standard
Detector TGS
Accumulation 16
Resolution 4 cm-1
Zero Filling On
Apodization Cosine
Gain Auto (1)
Aperture Auto (7.1 mm)
Scanning Speed Auto (2 mm/sec)
Filter Auto (30000 Hz)

Figure S9. IR spectrum of compound **1**

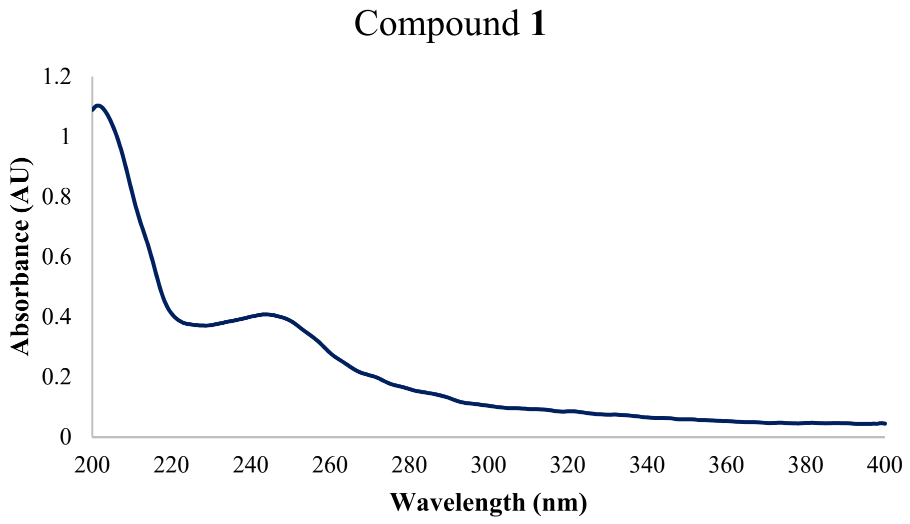

Figure S10. UV spectrum of compound **1**

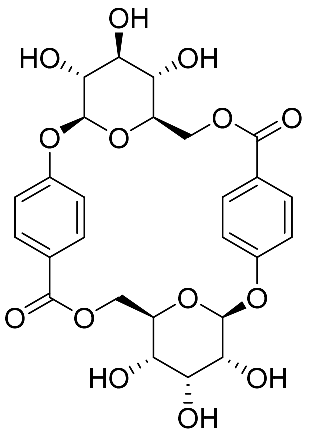

Structure of Compound **2**

Single Mass Analysis
Tolerance = 5.0 mDa / DBE: min = -1.5, max = 50.0
Element prediction: Off
Number of isotope peaks used for i-FIT = 3
Monoisotopic Mass, Even Electron Ions
161 formula(e) evaluated with 2 results within limits (up to 50 best isotopic matches for each mass)
Elements Used:

Mass	Calc. Mass	mDa	PPM	DBE	Formula	i-FIT	i-FIT Norm	Fit Conf %	C	H	O
563.1403	563.1401	0.2	0.4	13.5	C26 H27 O14	183.3	0.050	95.10	26	27	14
	563.1436	-3.3	-5.9	35.5	C44 H19 O	186.2	3.017	4.90	44	19	1

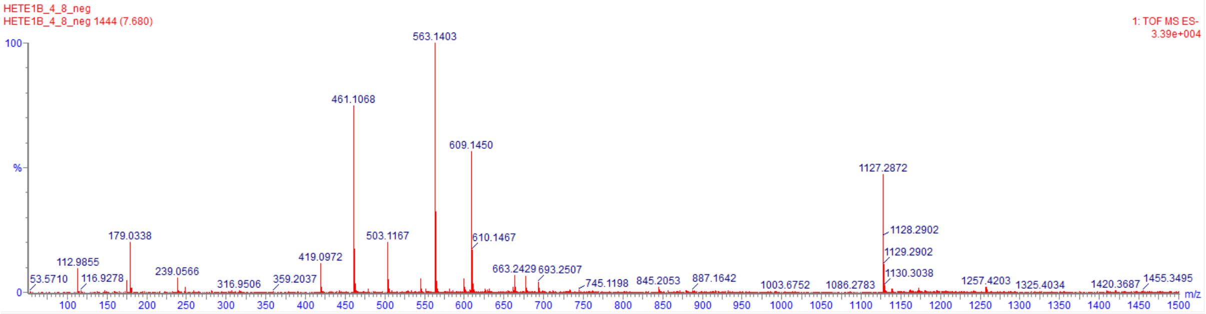

Figure S11. HRESI(−)MS spectrum of compound **2**

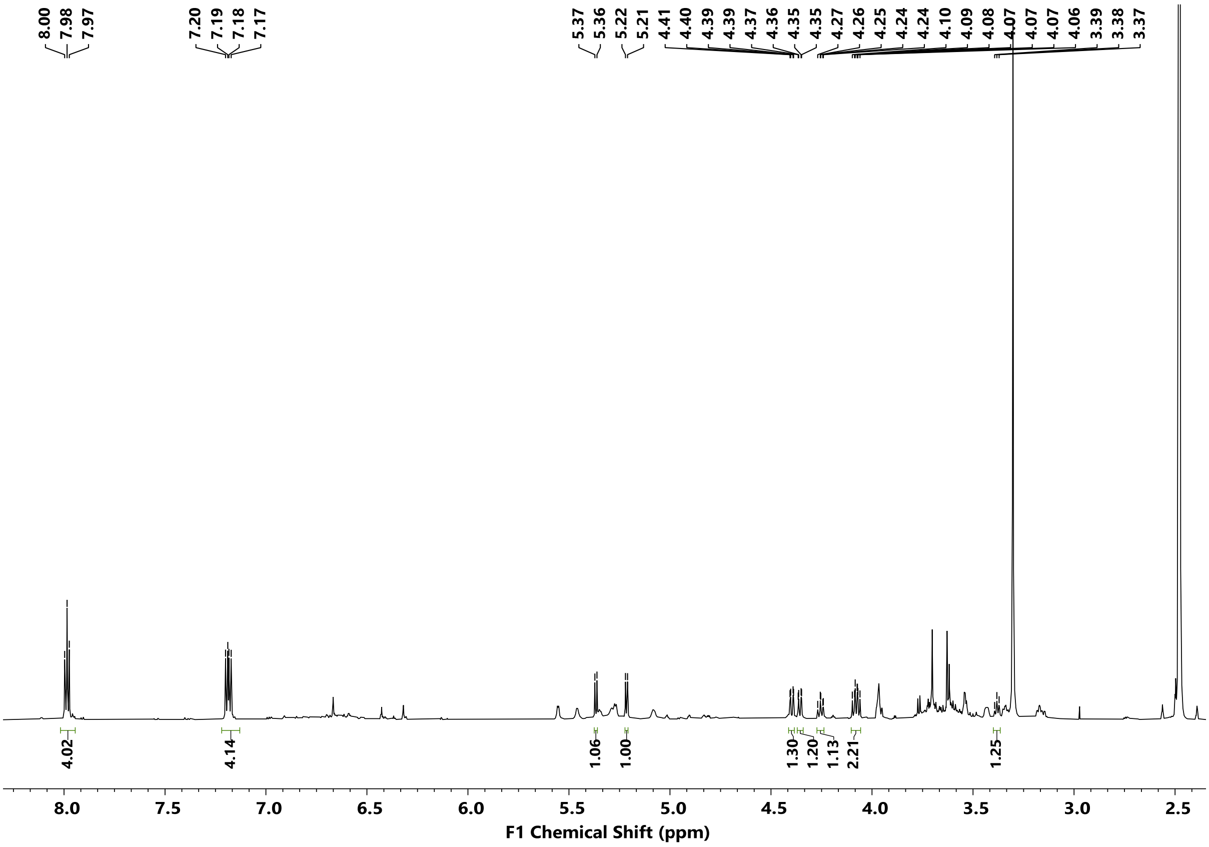

Figure S12. ^{1}H NMR spectrum of compound **2** in DMSO-d_6

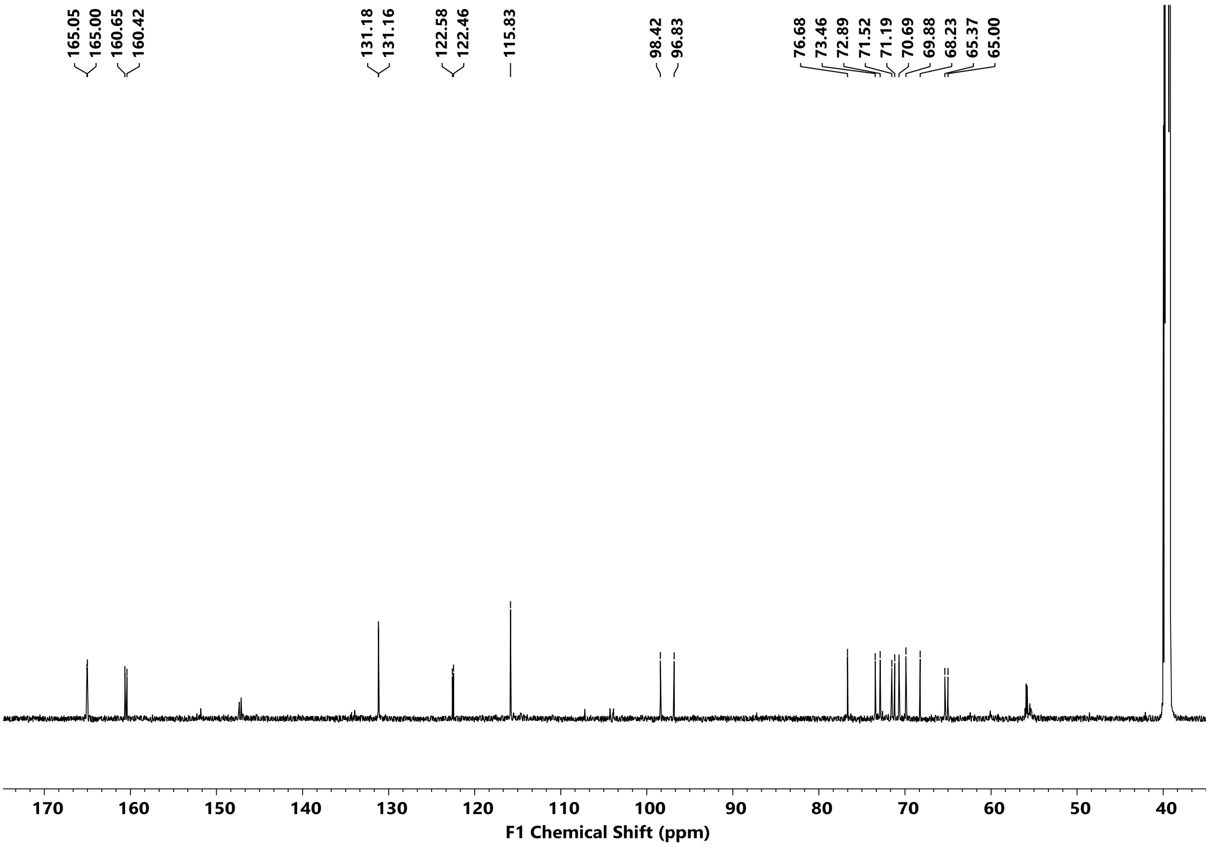

Figure S13. ^{13}C NMR spectrum of compound **2** in DMSO-d_6

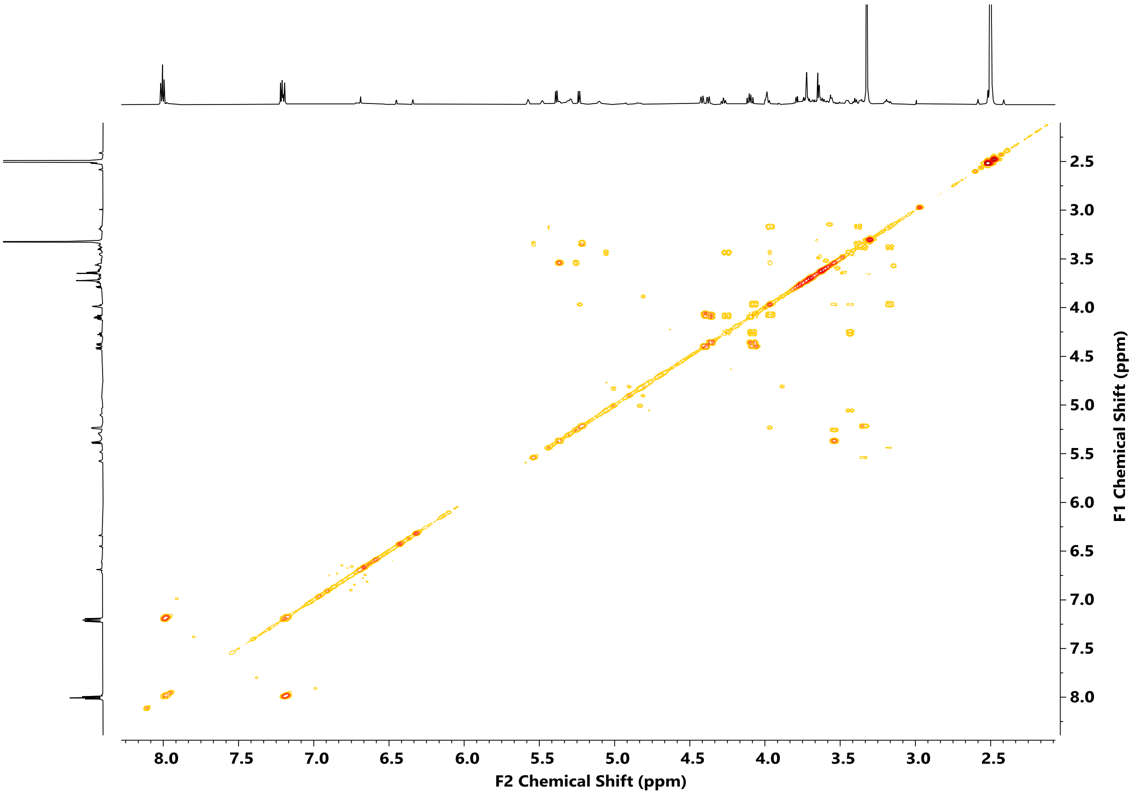

Figure S14. ^{1}H–^{1}H COSY spectrum of compound **2** in DMSO-d_6

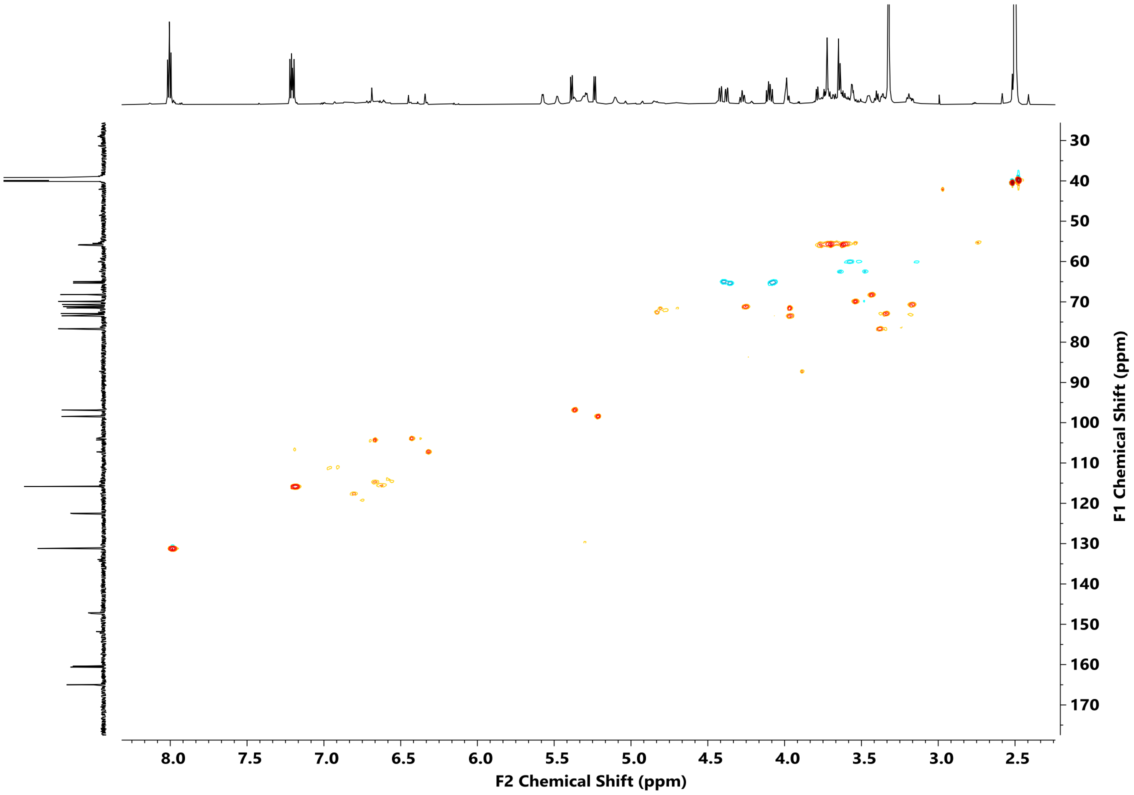

Figure S15. HSQC spectrum of compound **2** in DMSO-d_6

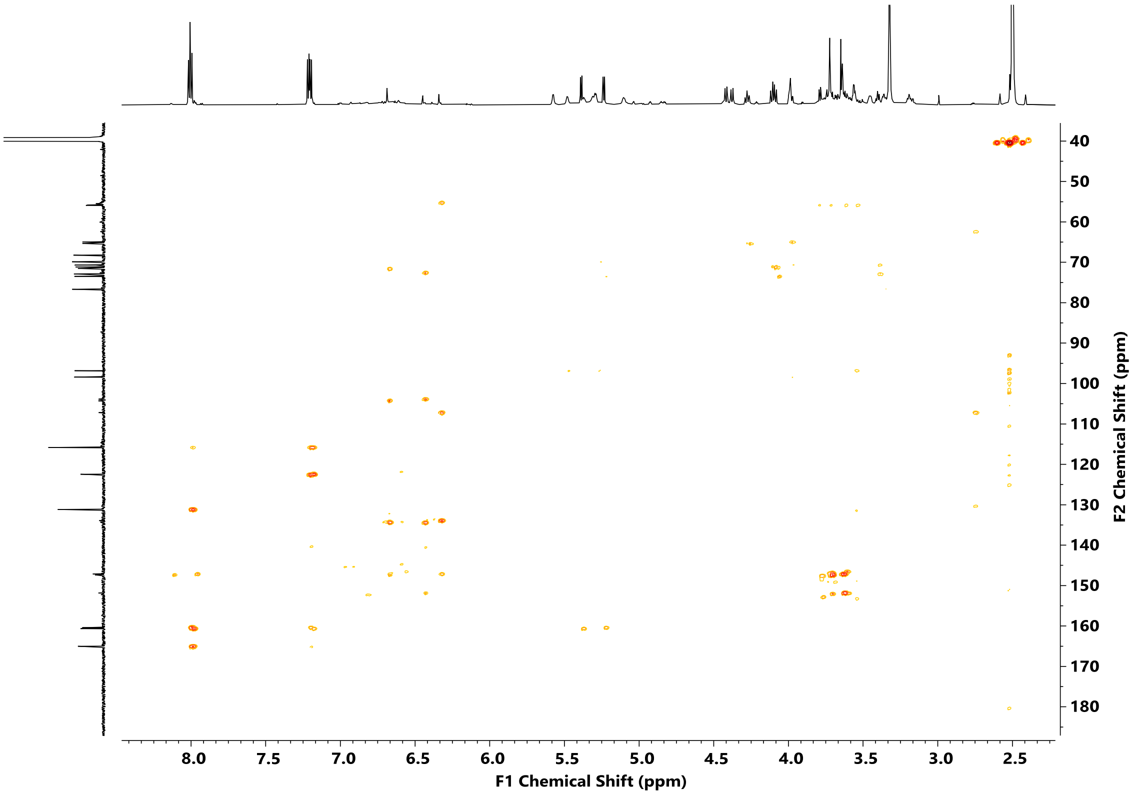

Figure 16. HMBC spectrum of compound **2** in DMSO-d_6

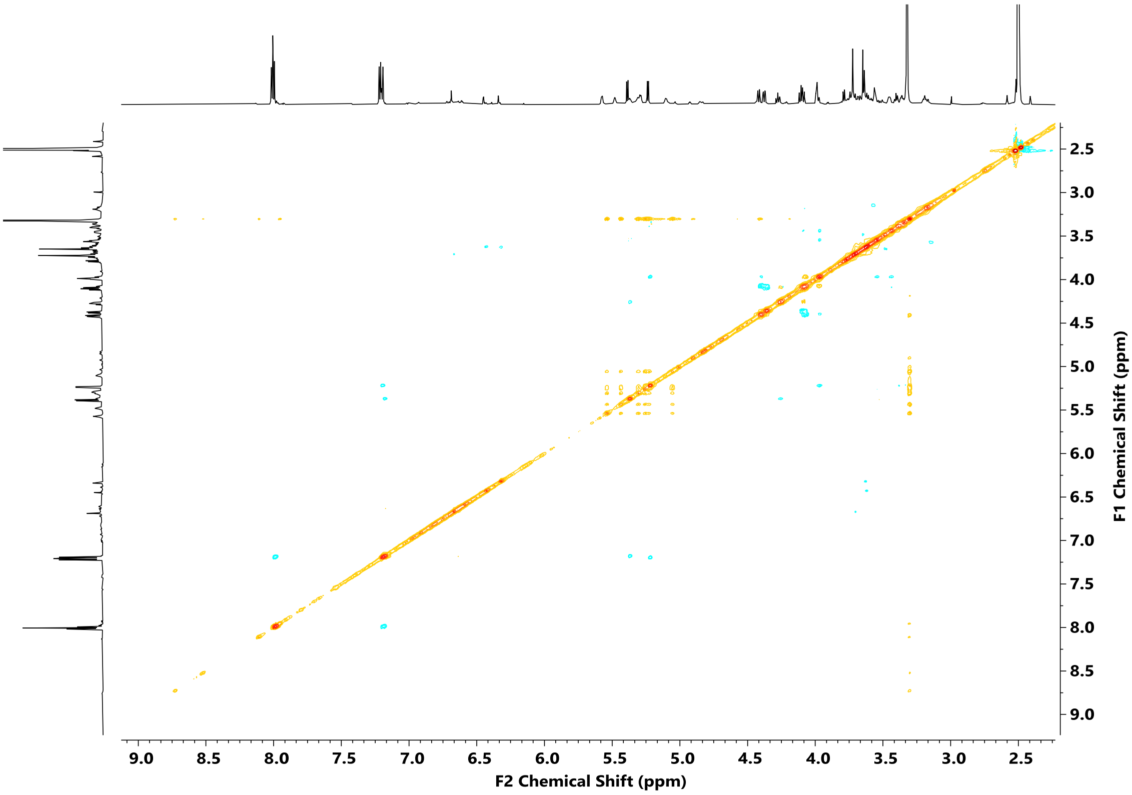

Figure S17. ROESY spectrum of compound **2** in DMSO-d_6

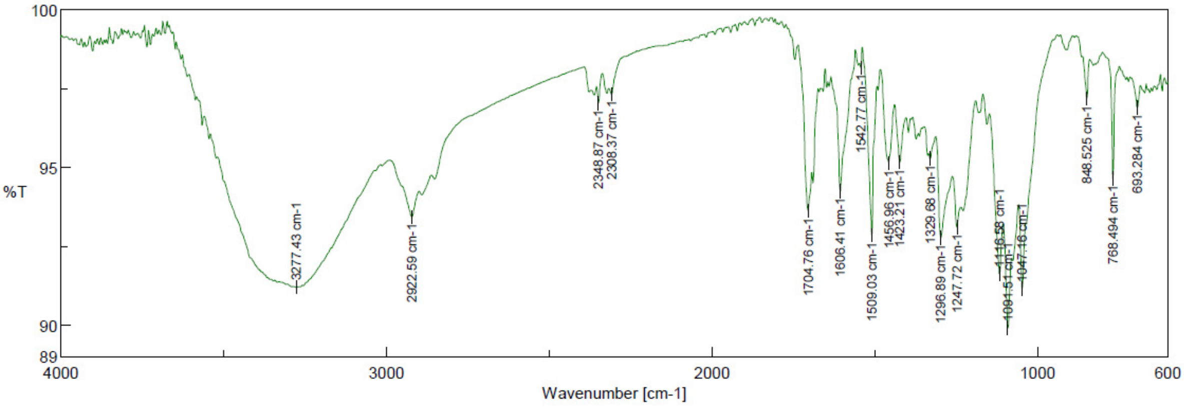

[Comment]
Sample Name 190729_HETE1E-S5
Comment
User
Division
Company 공동기기실

[Data Information]
Creation Date 2019-09-26 오후 4:28
Data array type Linear data array
Horizontal Wavenumber [cm-1]
Vertical %T
Start 599.753 cm-1
End 4000.6 cm-1
Data pitch 0.964233 cm-1
Data points 3528

[Measurement Information]
Model Name FT/IR-4200typeA
Serial Number B038361018

Light Source Standard
Detector TGS
Accumulation 16
Resolution 4 cm-1
Zero Filling On
Apodization Cosine
Gain Auto (2)
Aperture Auto (7.1 mm)
Scanning Speed Auto (2 mm/sec)
Filter Auto (30000 Hz)

Figure S18. IR spectrum of compound **2**

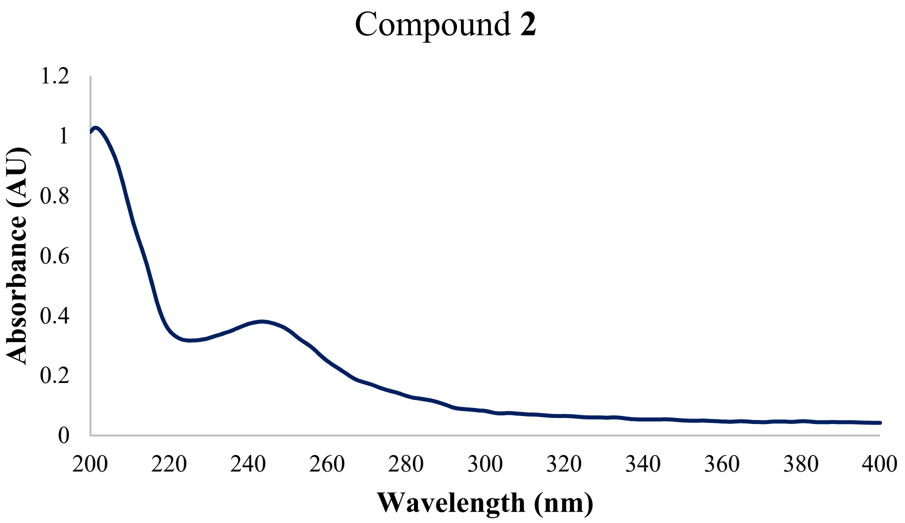

Figure S19. UV spectrum of compound **2**

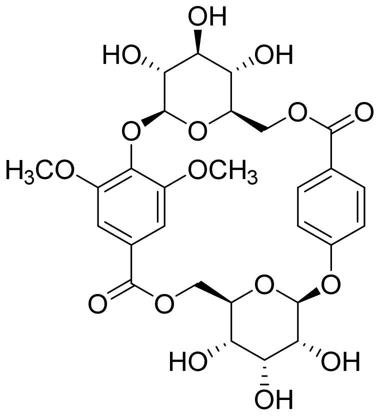

Structure of Compound **3**

Single Mass Analysis
Tolerance = 5.0 mDa / DBE: min = -1.5, max = 50.0
Element prediction: Off
Number of isotope peaks used for i-FIT = 3
Monoisotopic Mass, Even Electron Ions
191 formula(e) evaluated with 2 results within limits (up to 50 best isotopic matches for each mass)
Elements Used:

Mass	Calc. Mass	mDa	PPM	DBE	Formula	i-FIT	i-FIT Norm	Fit Conf %	C	H	O
623.1608	623.1612	-0.4	-0.6	13.5	C28 H31 O16	112.6	0.000	100.00	28	31	16
	623.1647	-3.9	-6.3	35.5	C46 H23 O3	127.0	14.385	0.00	46	23	3

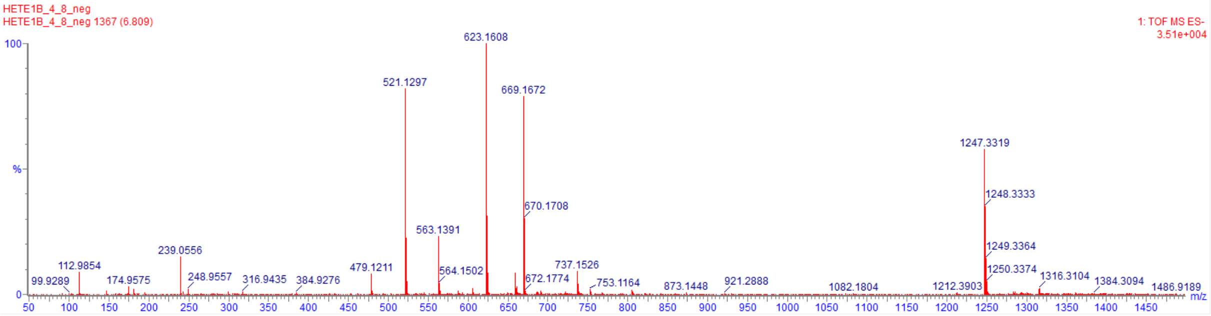

Figure S20. HRESI(−)MS spectrum of compound **3**

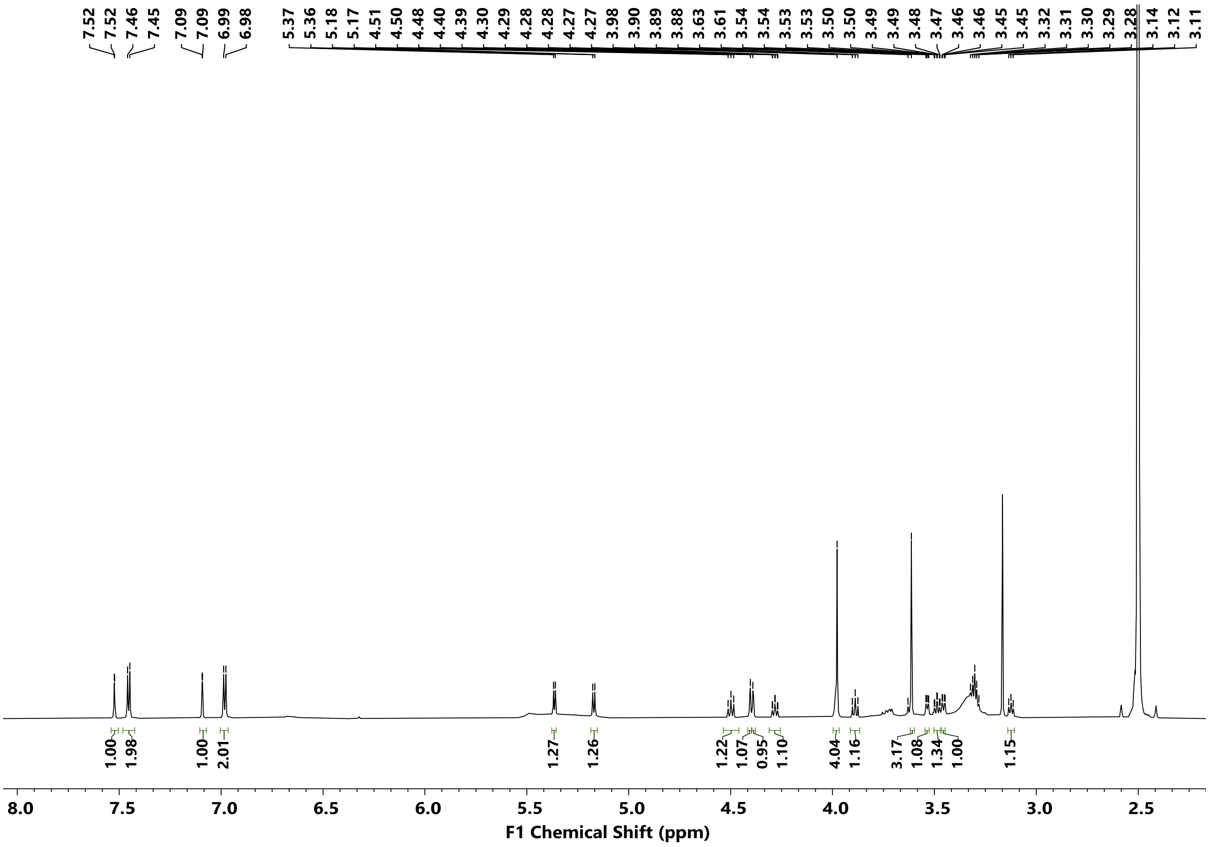

Figure S21. ^{1}H NMR spectrum of compound **3** in DMSO-d_6

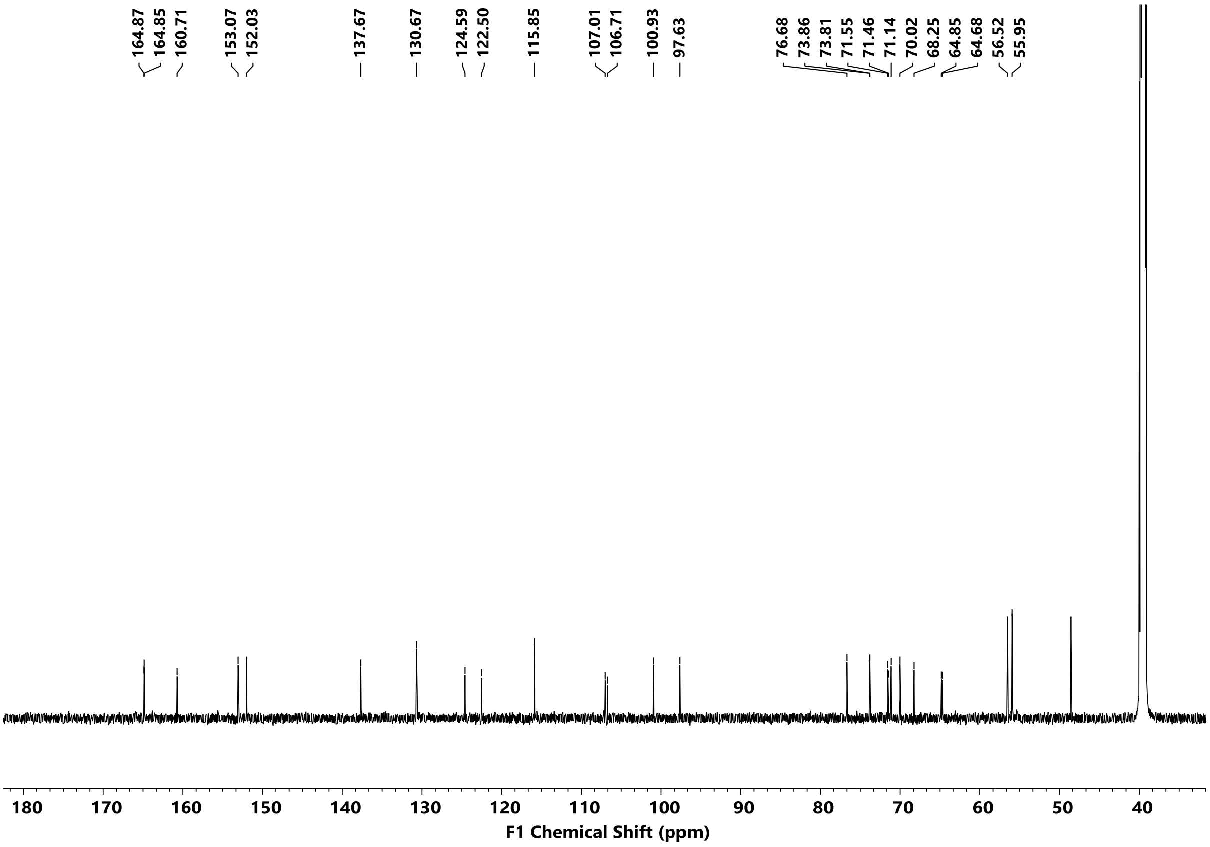

Figure S22. ^{13}C NMR spectrum of compound **3** in DMSO-d_6

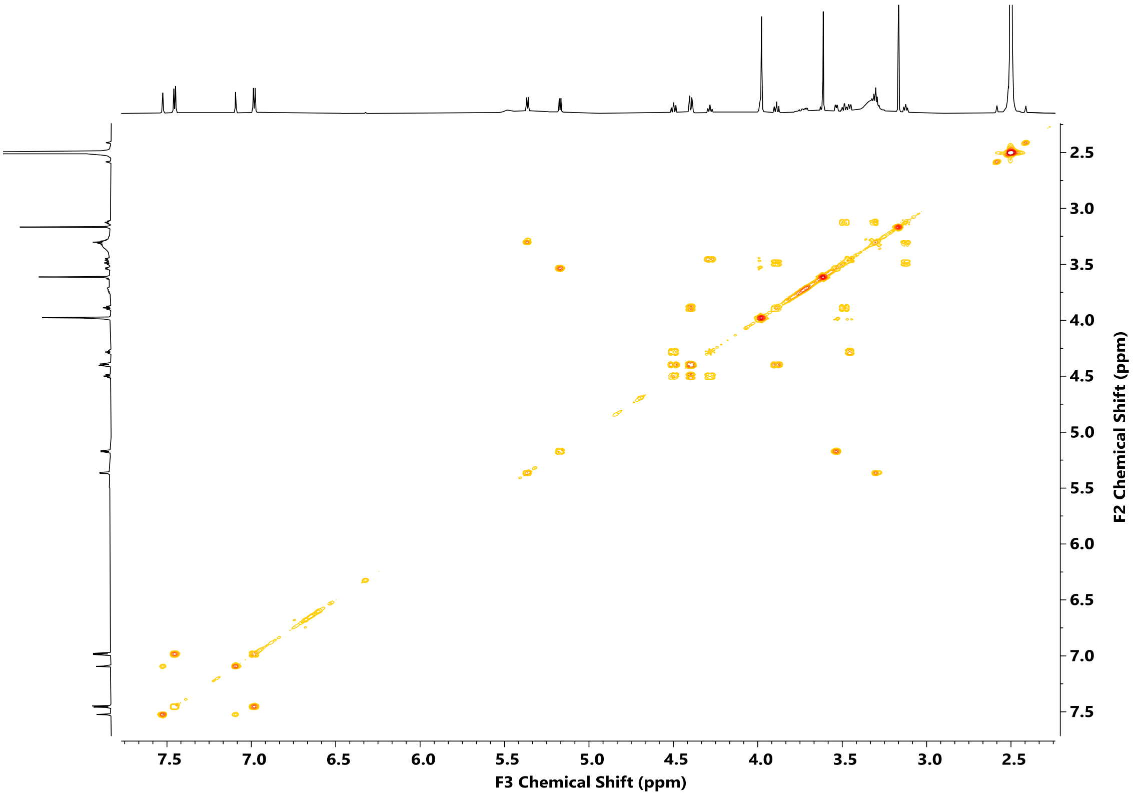

Figure S23. ^{1}H–^{1}H COSY spectrum of compound **3** in DMSO-*d*6

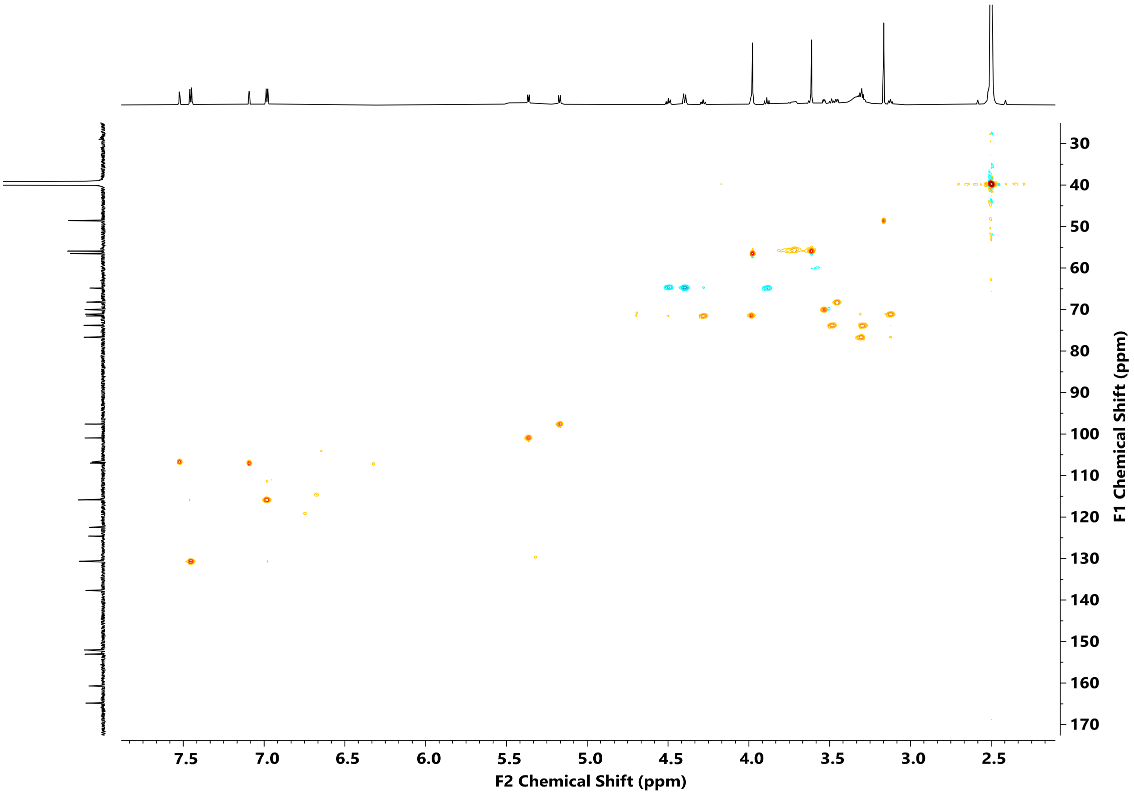

Figure S24. HSQC spectrum of compound **3** in DMSO-*d*6

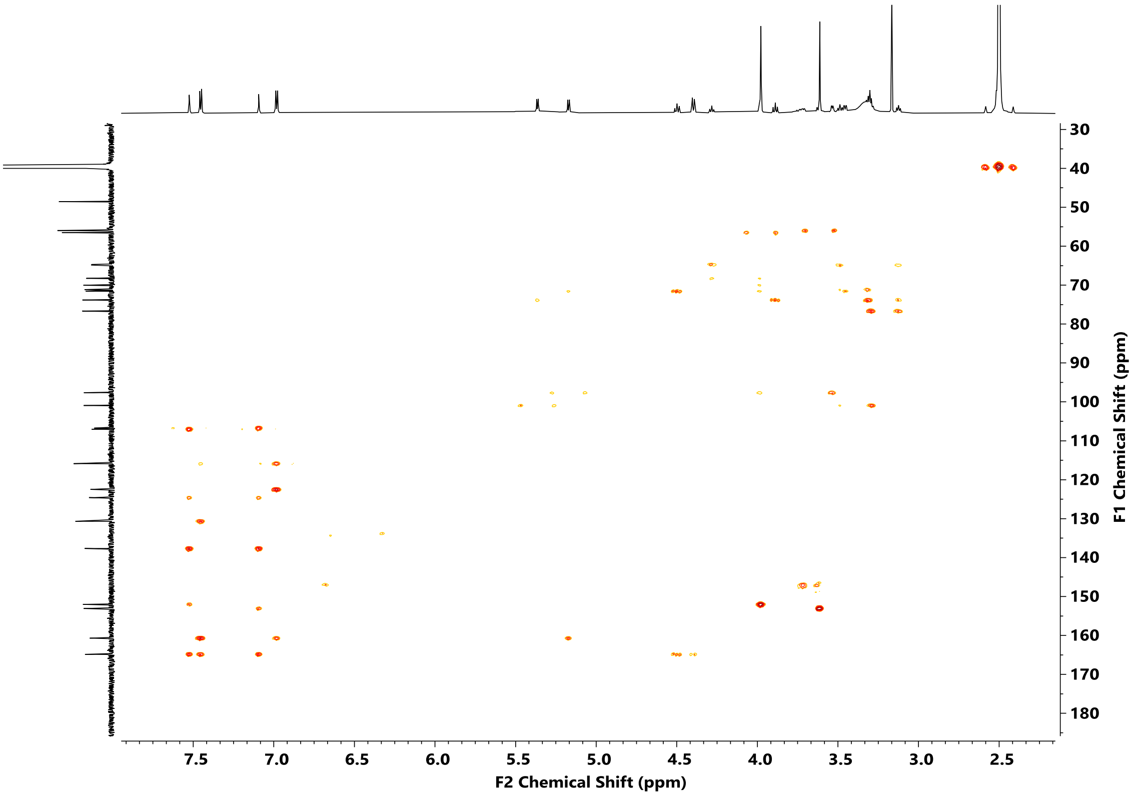

Figure S25. HMBC spectrum of compound **3** in DMSO-d_6

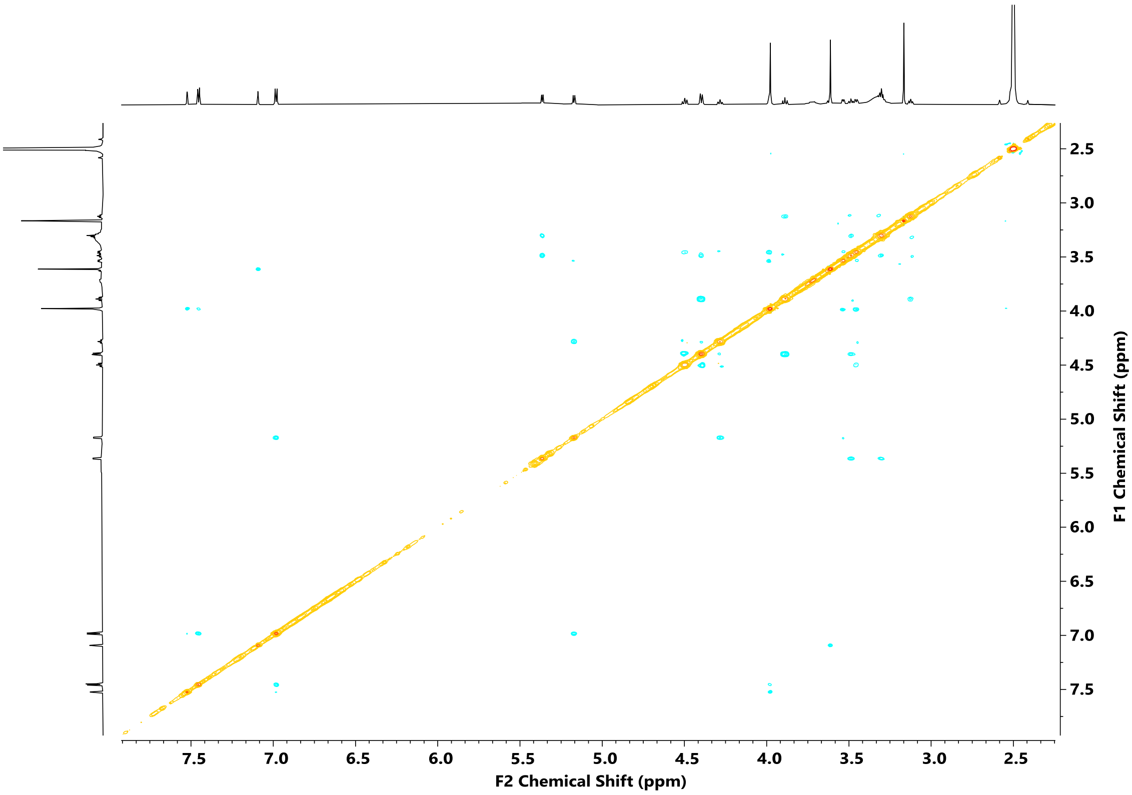

Figure S26. ROESY spectrum of compound **3** in DMSO-d_6

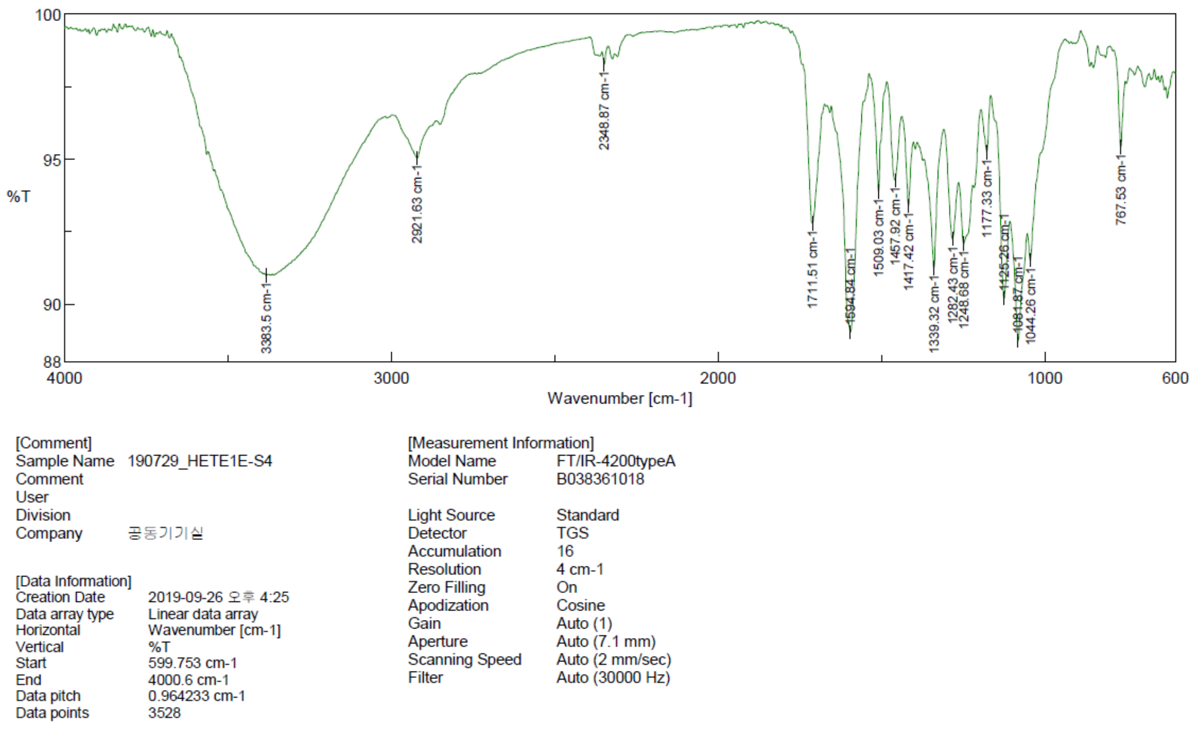

Figure S27. IR spectrum of compound **3**

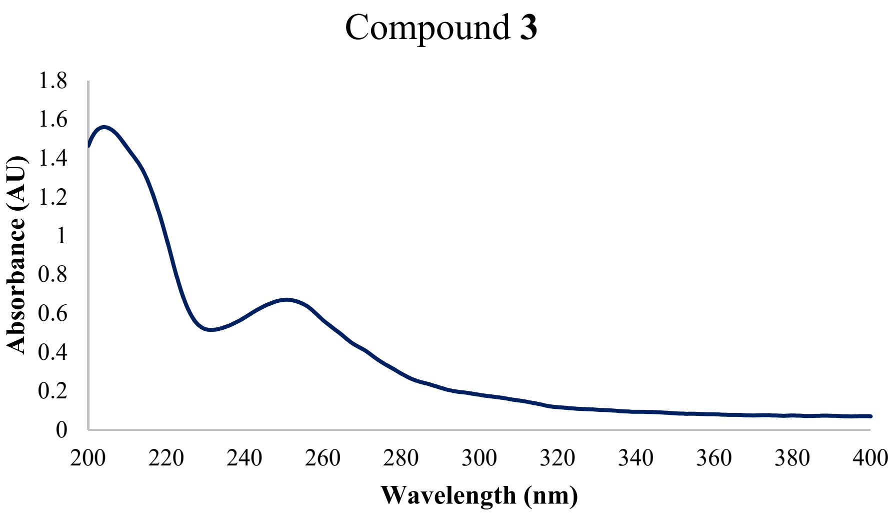

Figure S28.UV spectrum of compound **3**

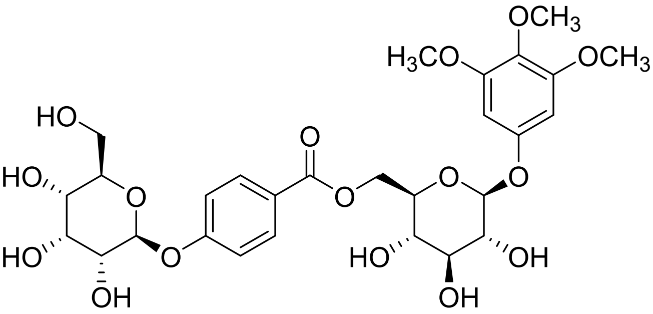

Structure of Compound **5**

Single Mass Analysis
Tolerance = 5.0 mDa / DBE: min = -1.5, max = 50.0
Element prediction: Off
Number of isotope peaks used for i-FIT = 3
Monoisotopic Mass, Even Electron Ions
195 formula(e) evaluated with 2 results within limits (up to 50 closest results for each mass)
Elements Used:

Mass	Calc. Mass	mDa	PPM	DBE	Formula	i-FIT	i-FIT Norm	Fit Conf %	C	H	O
627.1907	627.1925	-1.8	-2.9	11.5	C28 H35 O16	31.1	0.101	90.40	28	35	16
	627.1866	4.1	6.5	20.5	C35 H31 O11	33.3	2.344	9.60	35	31	11

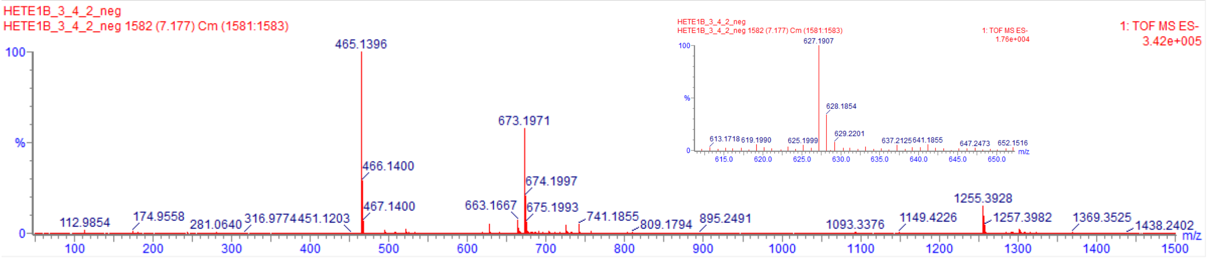

Figure S29. HRESI(−)MS spectrum of compound **5**

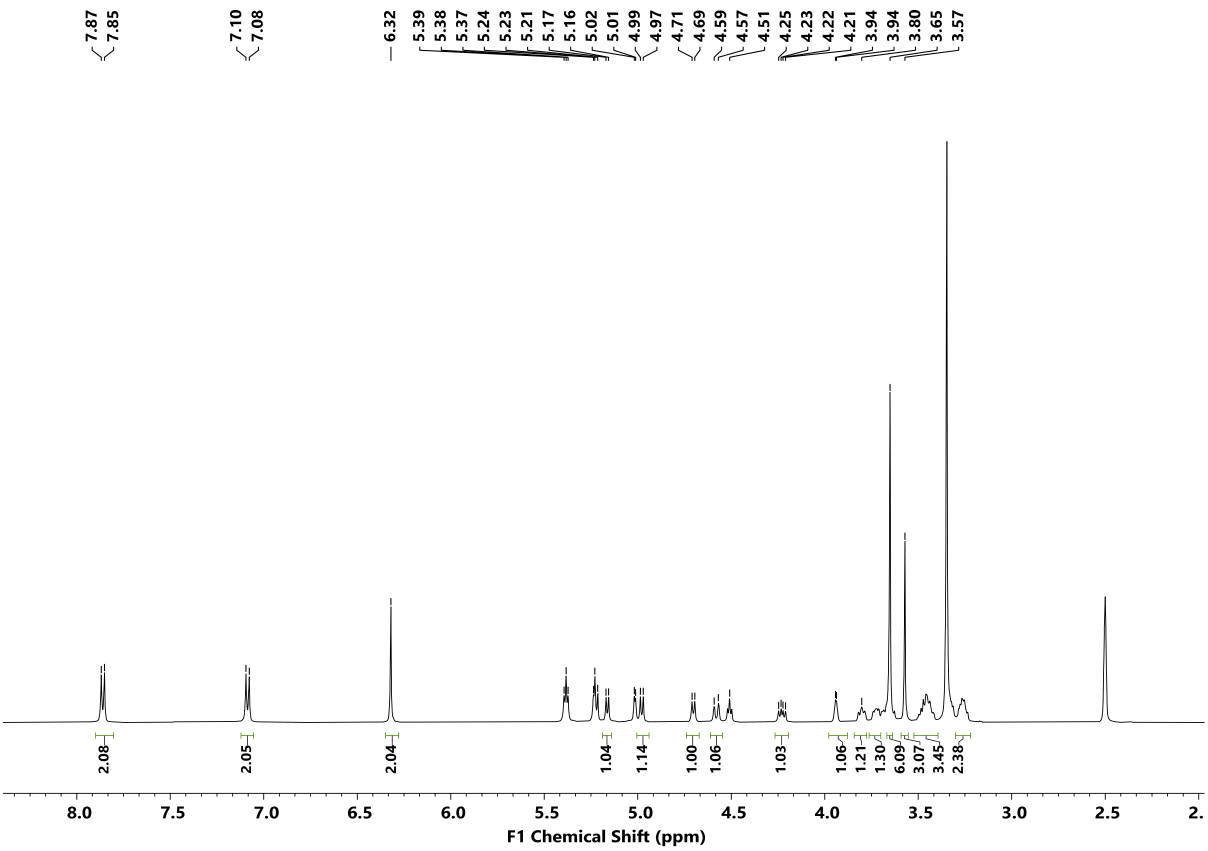

Figure S30. ^{1}H NMR spectrum of compound **5** in DMSO-d_6

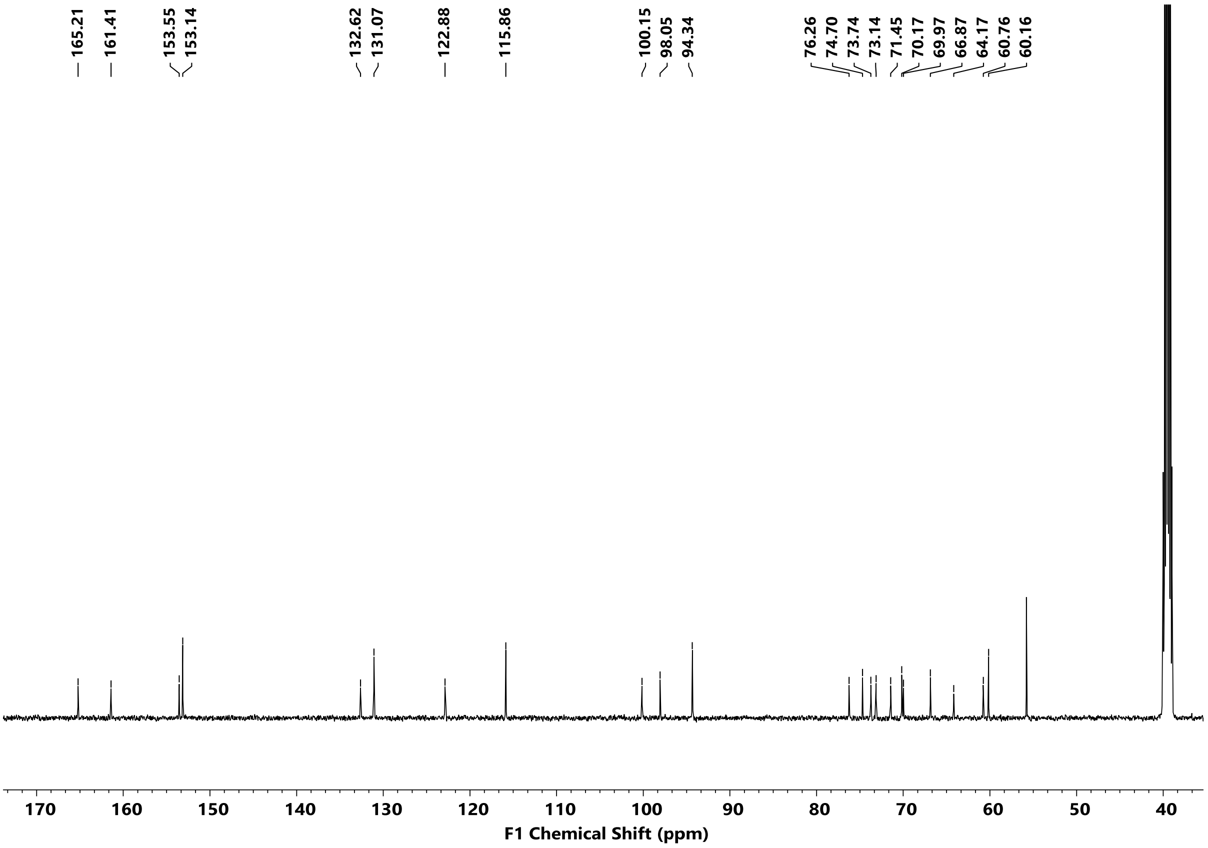

Figure S31. ^{13}C NMR spectrum of compound **5** in DMSO-d_6

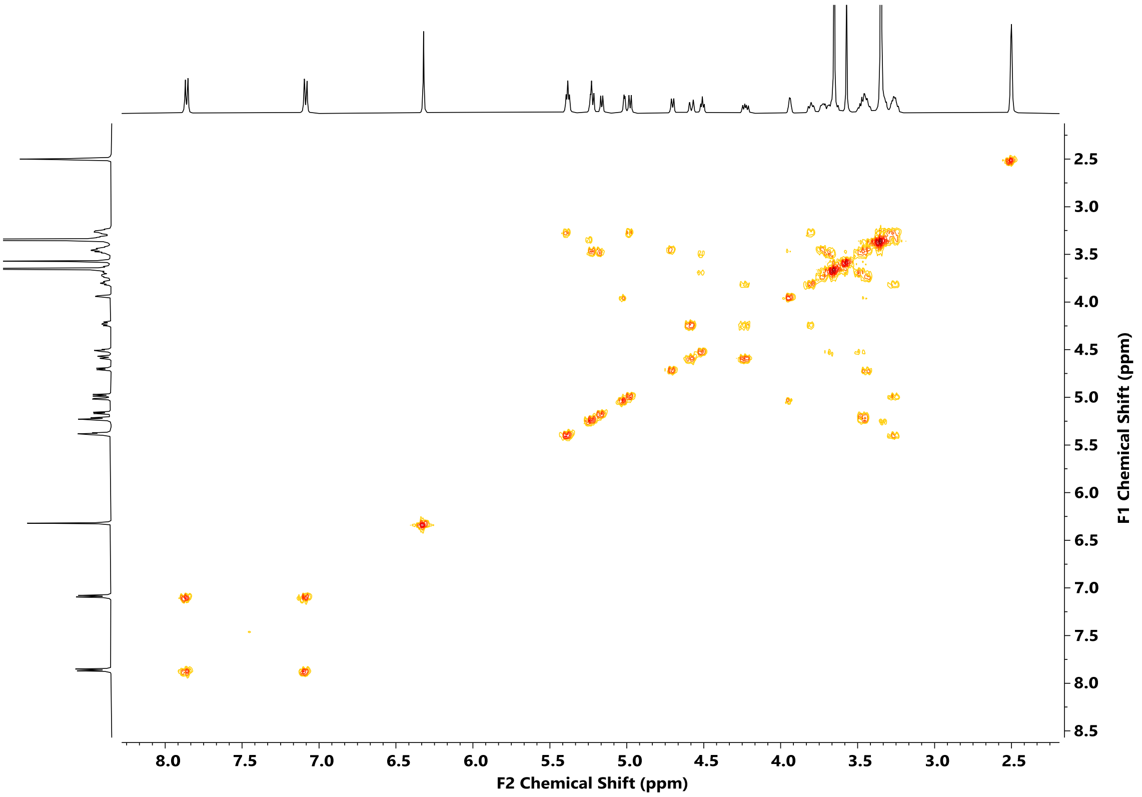

Figure S32. $^{1}H-^{1}H$ COSY spectrum of compound **5** in DMSO-d_6

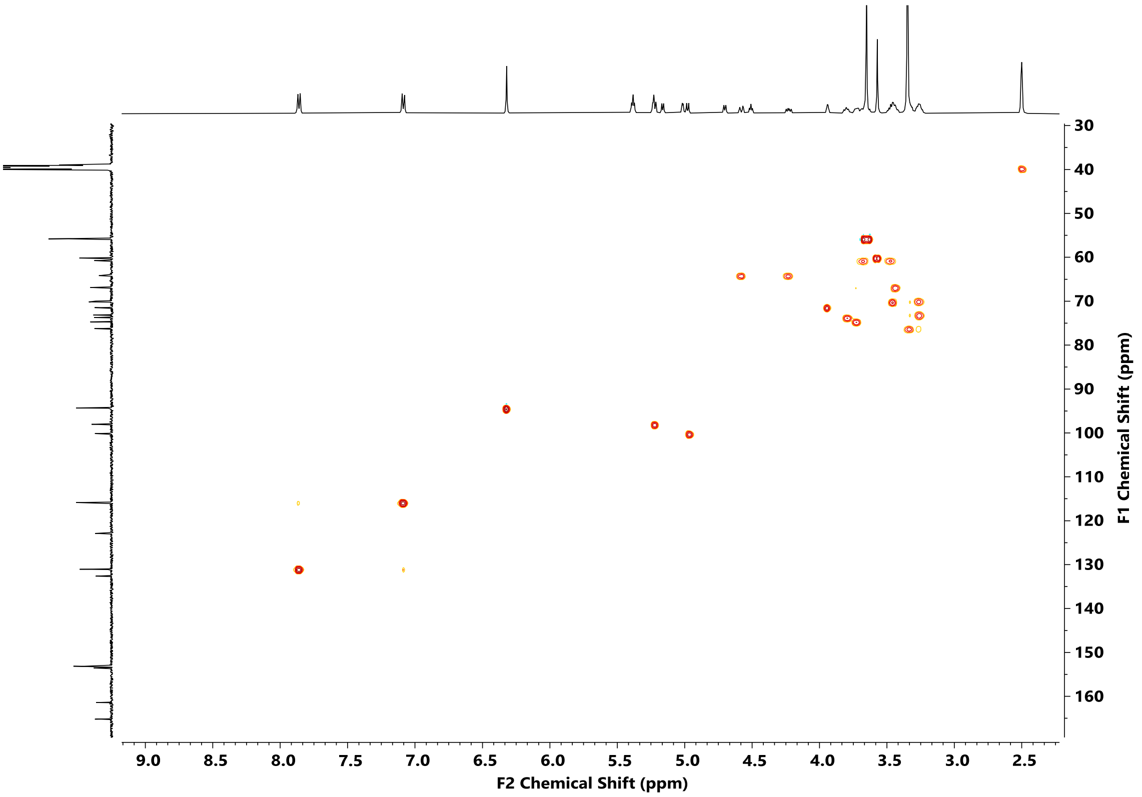

Figure S33. HSQC spectrum of compound **5** in DMSO-d_6

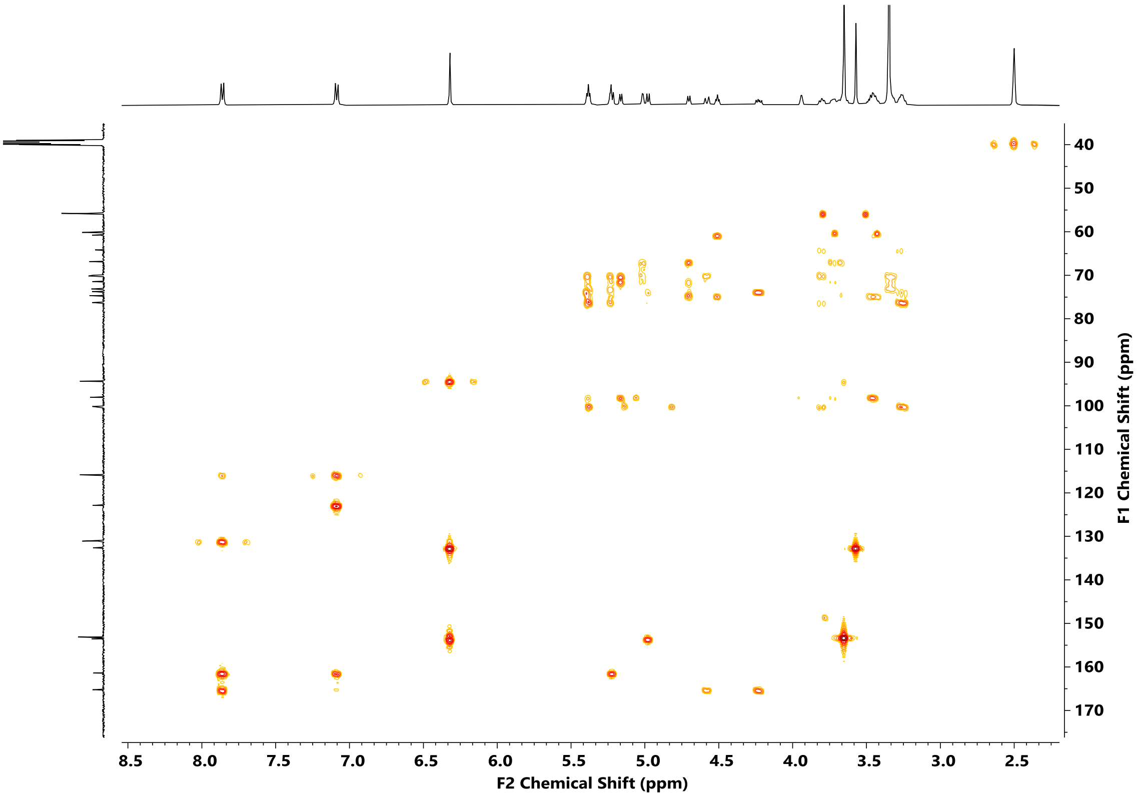

Figure S34. HMBC spectrum of compound **5** in DMSO-d_6

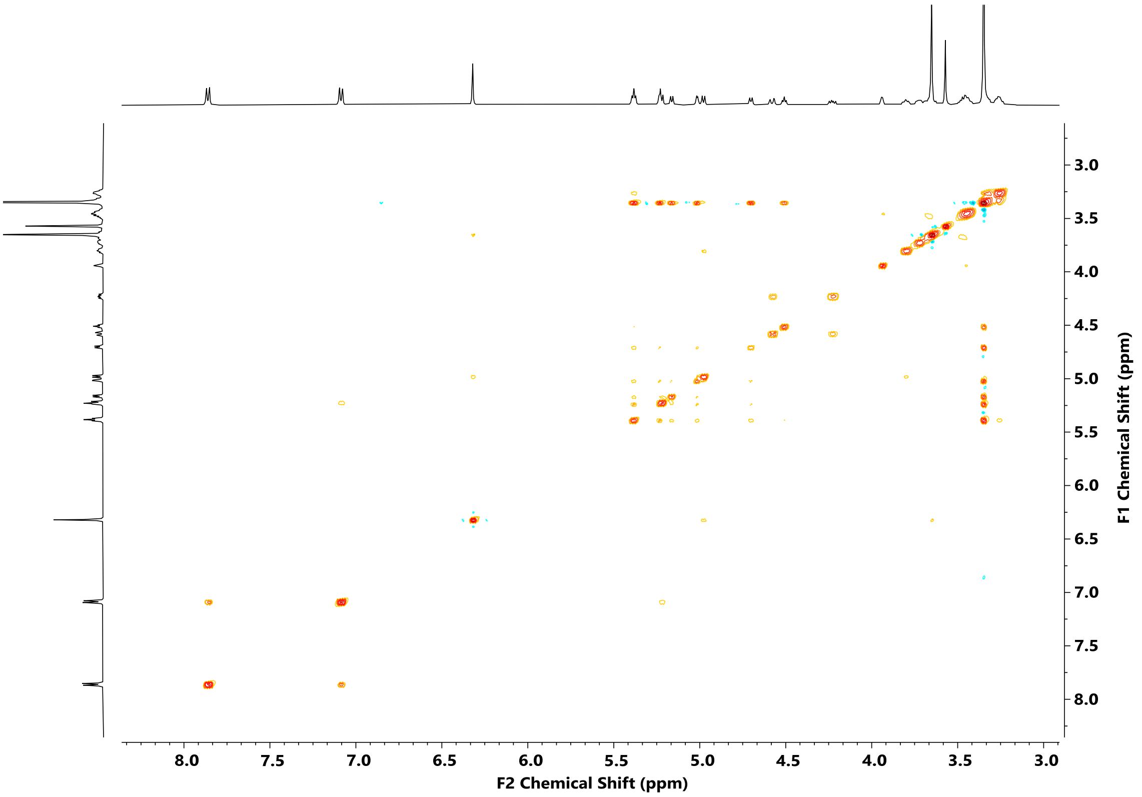

Figure S35. ROESY spectrum of compound **5** in DMSO-d_6

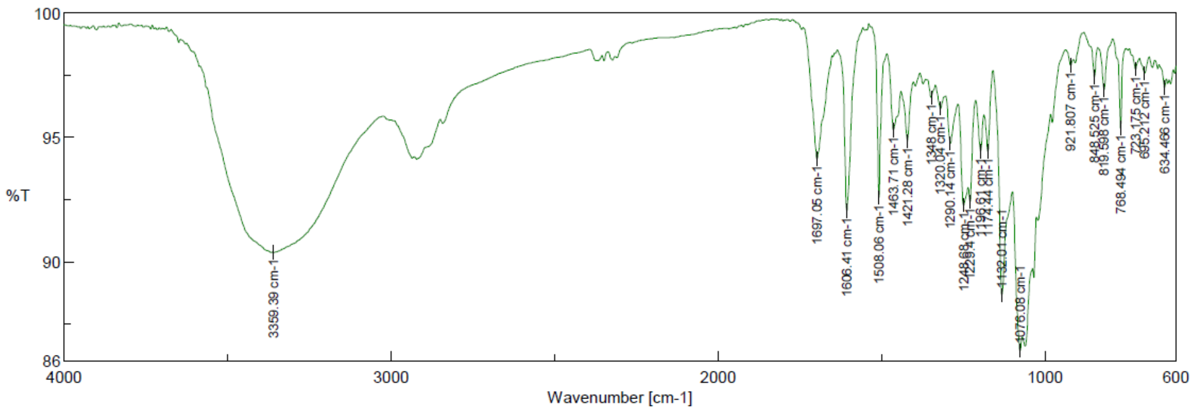

[Comment]
Sample Name 190729_HETE1E-S3
Comment
User
Division
Company 공동기기실

[Data Information]
Creation Date 2019-07-29 오후 5:06
Data array type Linear data array
Horizontal Wavenumber [cm-1]
Vertical %T
Start 599.753 cm-1
End 4000.6 cm-1
Data pitch 0.964233 cm-1
Data points 3528

[Measurement Information]
Model Name FT/IR-4200typeA
Serial Number B038361018

Light Source Standard
Detector TGS
Accumulation 16
Resolution 4 cm-1
Zero Filling On
Apodization Cosine
Gain Auto (2)
Aperture Auto (7.1 mm)
Scanning Speed Auto (2 mm/sec)
Filter Auto (30000 Hz)

Figure S36. IR spectrum of compound **5**

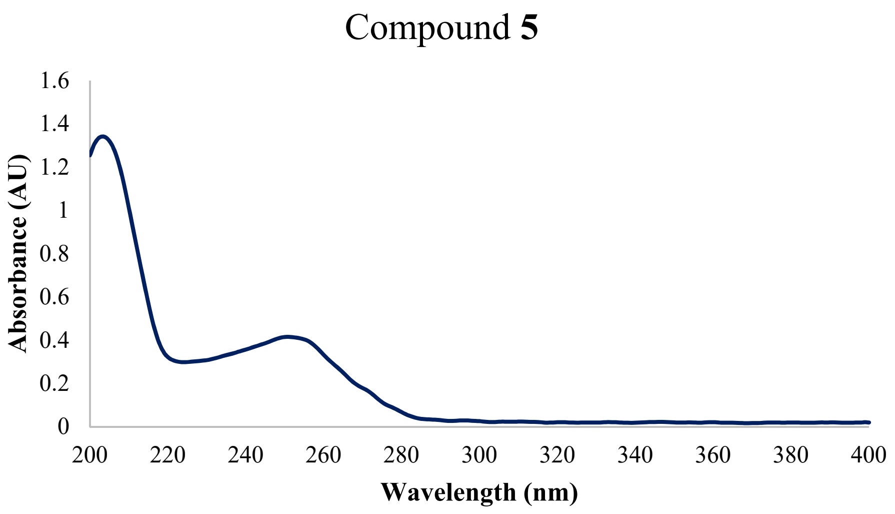

Figure S37. UV spectrum of compound **5**

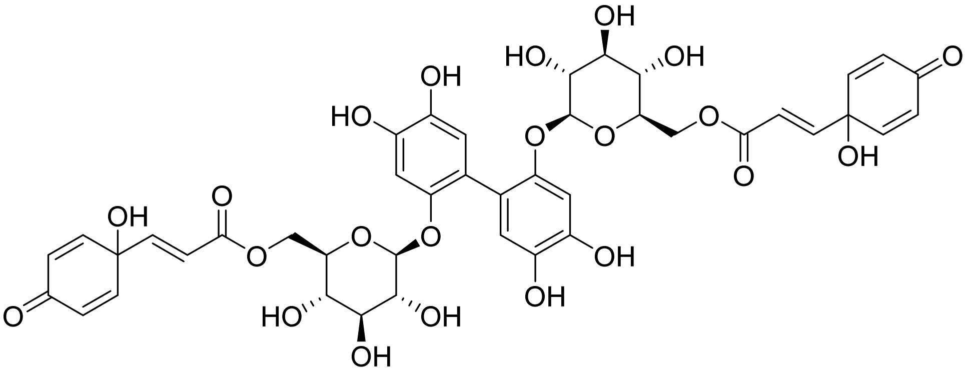

Structure of Compound **6**

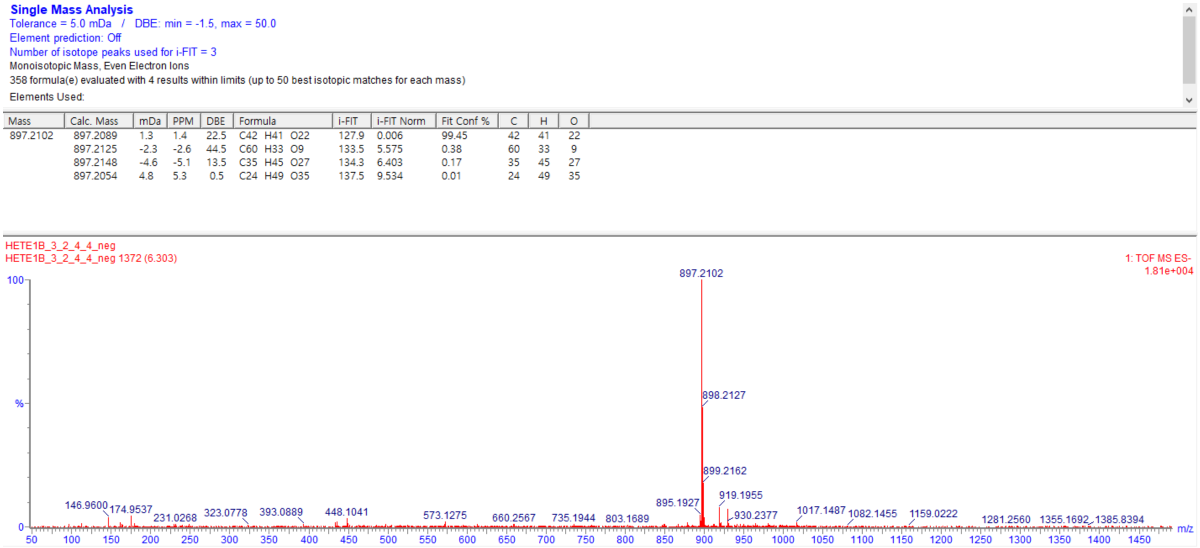

Single Mass Analysis
Tolerance = 5.0 mDa / DBE: min = -1.5, max = 50.0
Element prediction: Off
Number of isotope peaks used for i-FIT = 3
Monoisotopic Mass, Even Electron Ions
358 formula(e) evaluated with 4 results within limits (up to 50 best isotopic matches for each mass)
Elements Used:

Mass	Calc. Mass	mDa	PPM	DBE	Formula	i-FIT	i-FIT Norm	Fit Conf %	C	H	O
897.2102	897.2089	1.3	1.4	22.5	C42 H41 O22	127.9	0.006	99.45	42	41	22
	897.2125	-2.3	-2.6	44.5	C60 H33 O9	133.5	5.575	0.38	60	33	9
	897.2148	-4.6	-5.1	13.5	C35 H45 O27	134.3	6.403	0.17	35	45	27
	897.2054	4.8	5.3	0.5	C24 H49 O35	137.5	9.534	0.01	24	49	35

Figure S38. HRESI(−)MS spectrum of compound **6**

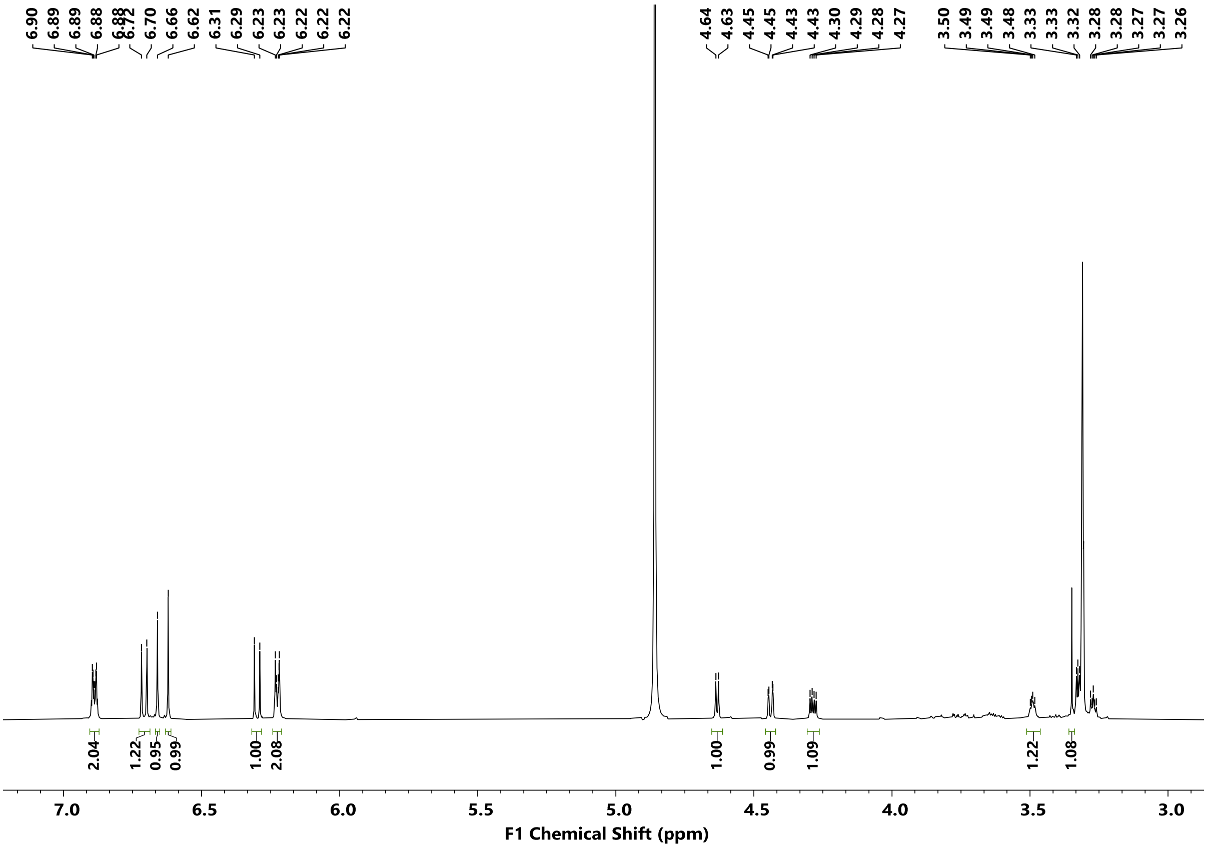

Figure S39. ^{1}H NMR spectrum of compound **6** in CD_3OD

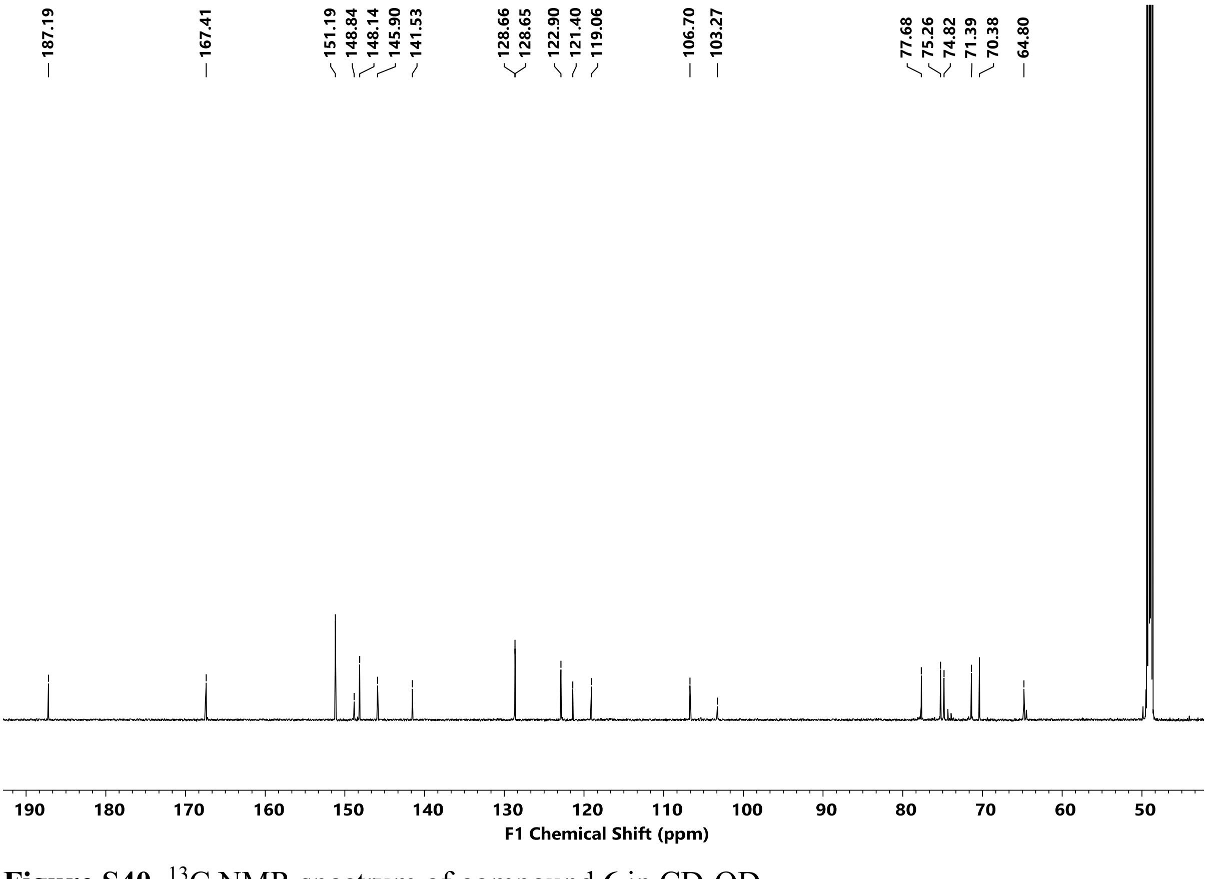

Figure S40. ^{13}C NMR spectrum of compound **6** in CD_3OD

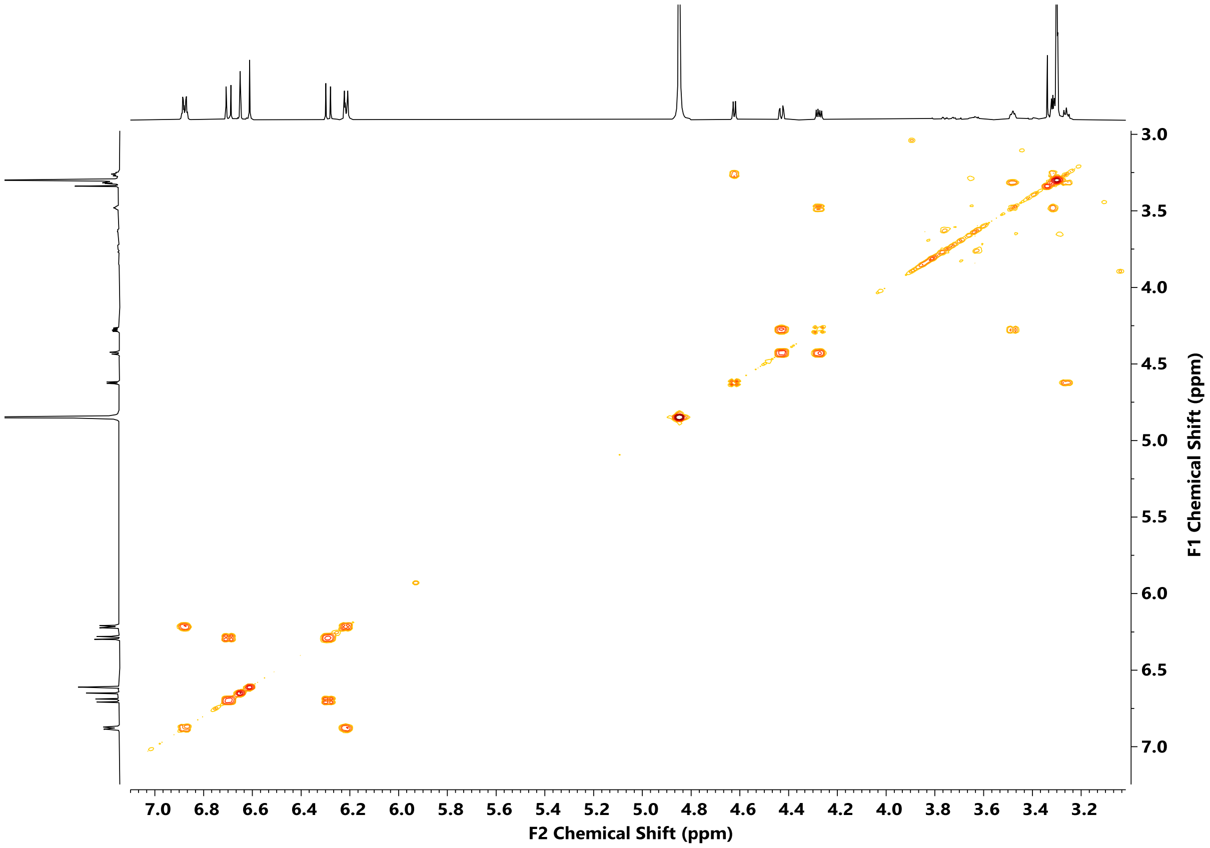

Figure S41. ^{1}H–^{1}H COSY spectrum of compound **6** in CD_3OD

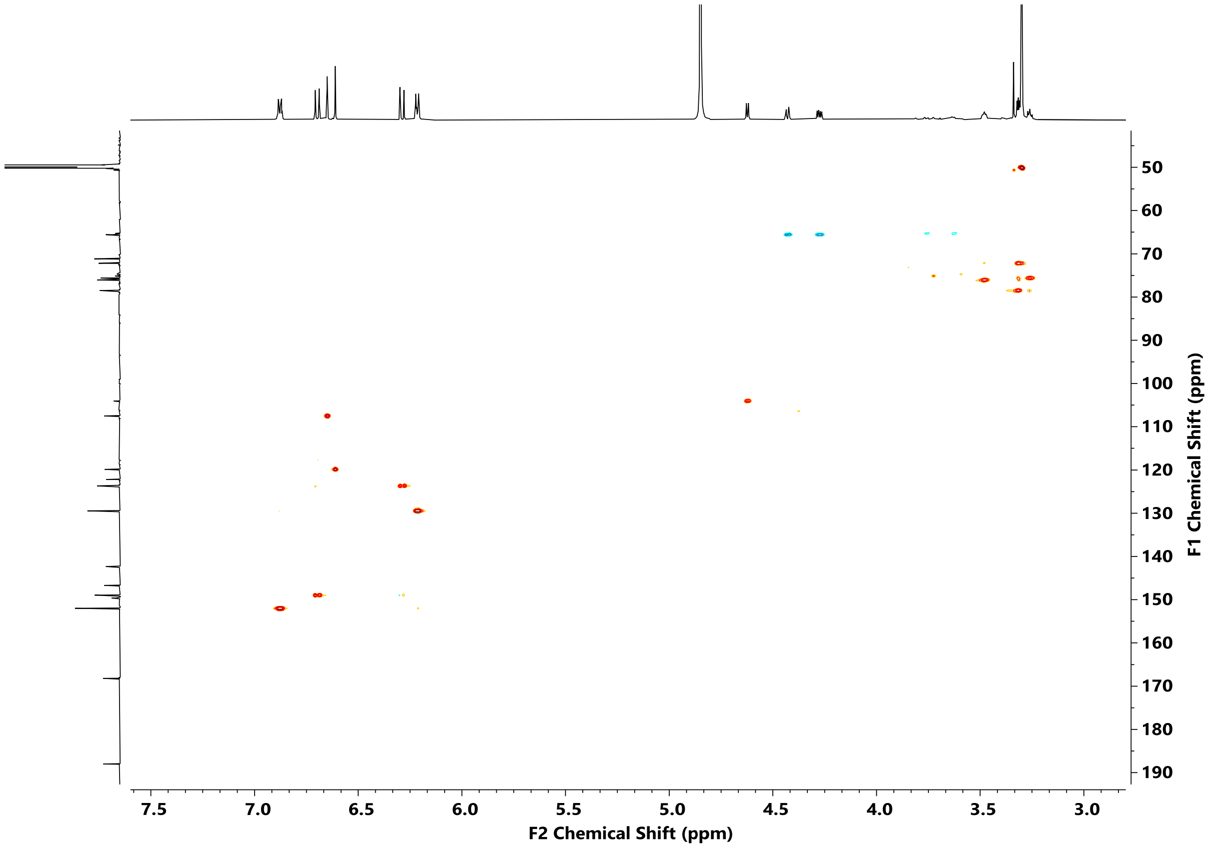

Figure S42. HSQC spectrum of compound **6** in CD_3OD

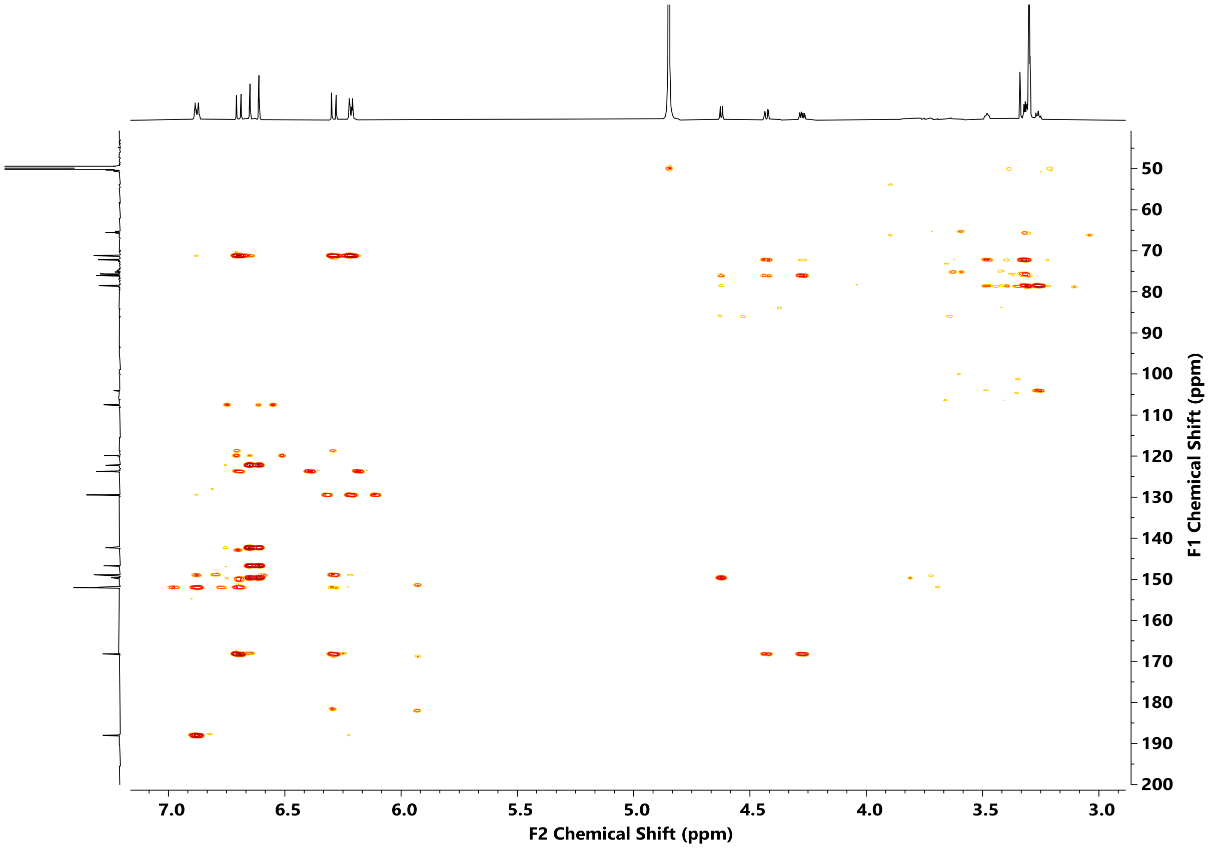

Figure S43. HMBC spectrum of compound **6** in CD_3OD

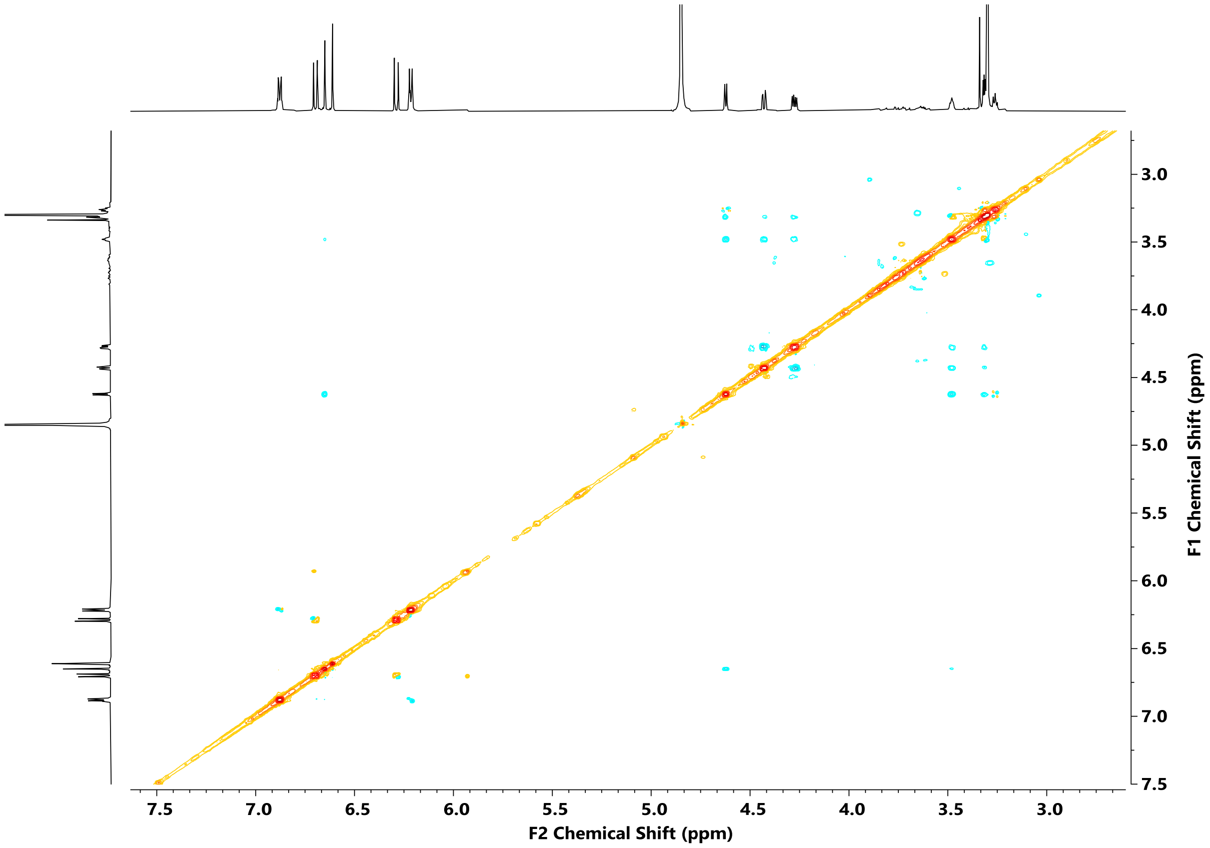

Figure S44. ROESY spectrum of compound **6** in CD_3OD

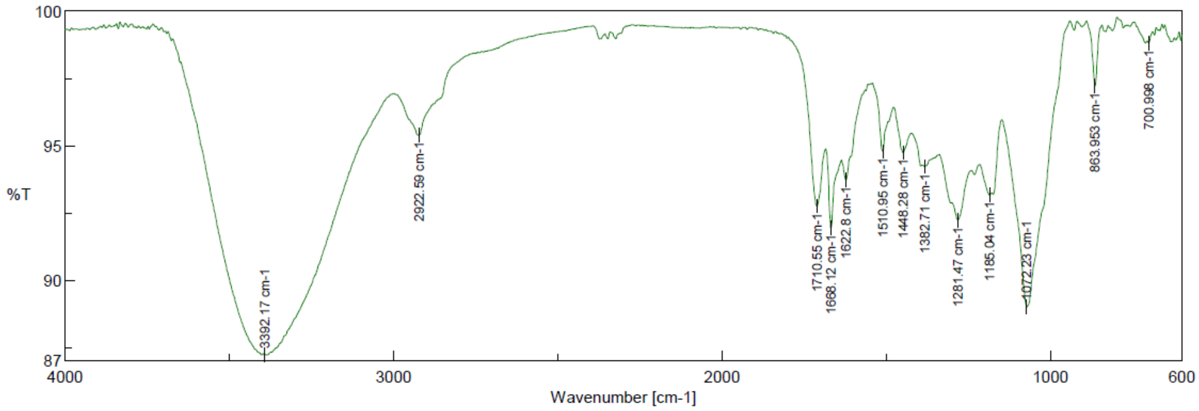

[Comment]
Sample Name 190729_HETE1E-S2
Comment
User
Division
Company 공동기기실

[Data Information]
Creation Date 2019-07-29 오후 4:56
Data array type Linear data array
Horizontal Wavenumber [cm-1]
Vertical %T
Start 599.753 cm-1
End 4000.6 cm-1
Data pitch 0.964233 cm-1
Data points 3528

[Measurement Information]
Model Name FT/IR-4200typeA
Serial Number B038361018

Light Source Standard
Detector TGS
Accumulation 16
Resolution 4 cm-1
Zero Filling On
Apodization Cosine
Gain Auto (1)
Aperture Auto (7.1 mm)
Scanning Speed Auto (2 mm/sec)
Filter Auto (30000 Hz)

Figure S45. IR spectrum of compound **6**

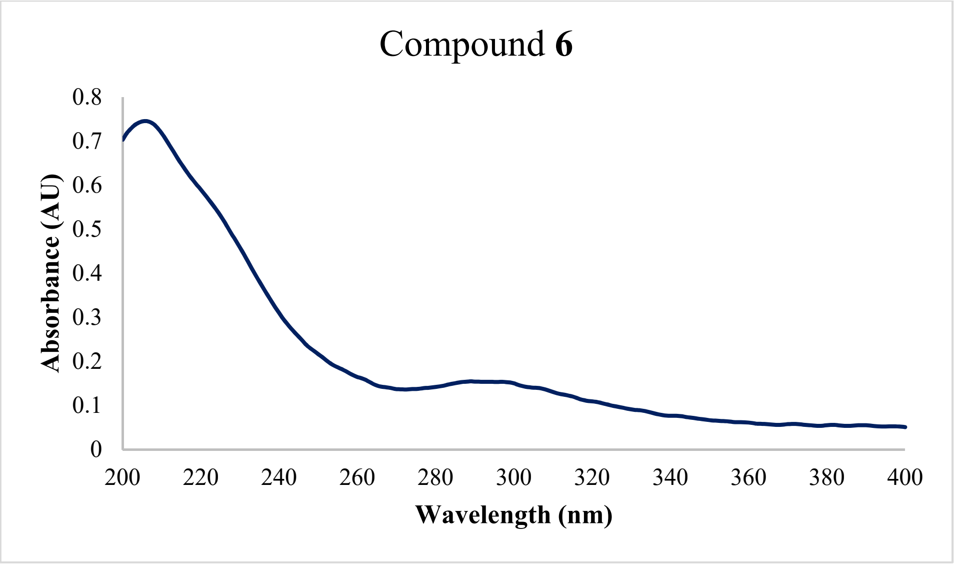

Figure S46. UV spectrum of compound **6**

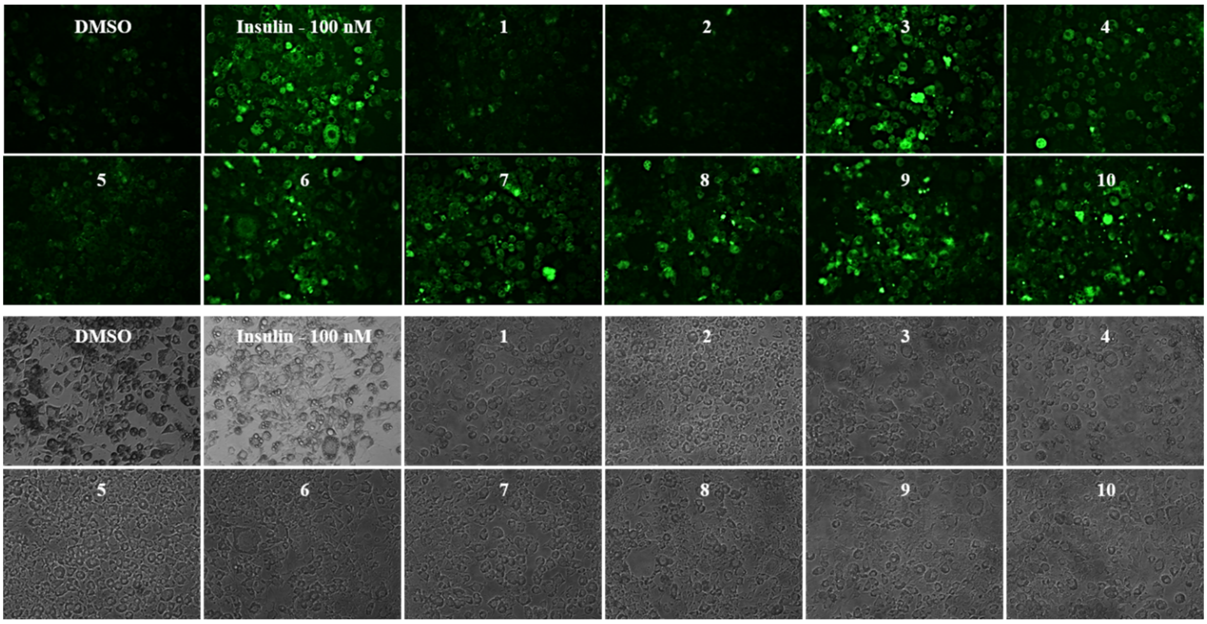

Figure S47. Effects of compounds **1-10** on 2-NBDG uptake in 3T3-L1 adipocytes. The fluorescence and bright-field images were captured by the fluorescence microscopy method.

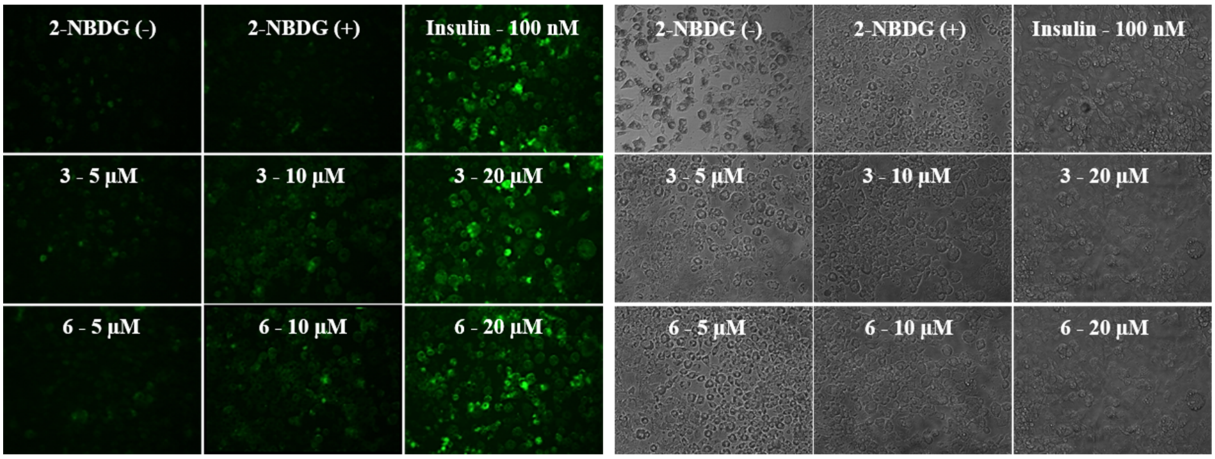

Figure S48. Stimulation effects of compounds **3** and **6** at different concentrations (5, 10 and 20 μM) on 2-NBDG uptake using 3T3-L1 adipocytes. The fluorescence and bright-field images were captured by the fluorescence microscopy method.

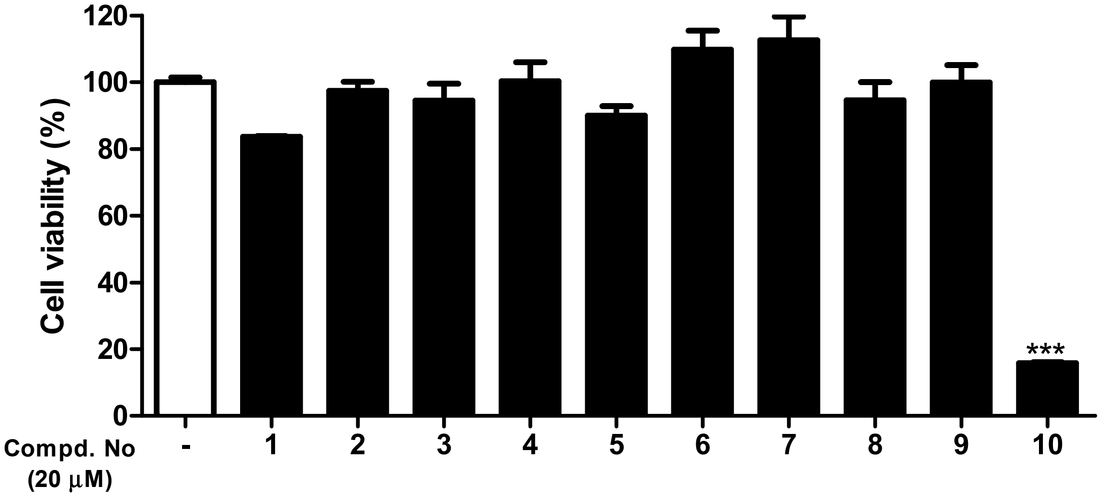

Figure S49. The cytotoxicity effects of compounds **1-10** at a concentration of 20 μM (A) in 3T3-L1 adipocytes. The cells were incubated with compounds for 24 h at 37°C. Then, the MTT assay was performed as described in the experimental section. The results were calculated as the mean ± SD ($n = 3$), * $p < 0.05$, ** $p < 0.01$ and *** $p < 0.001$, compared to the negative control.

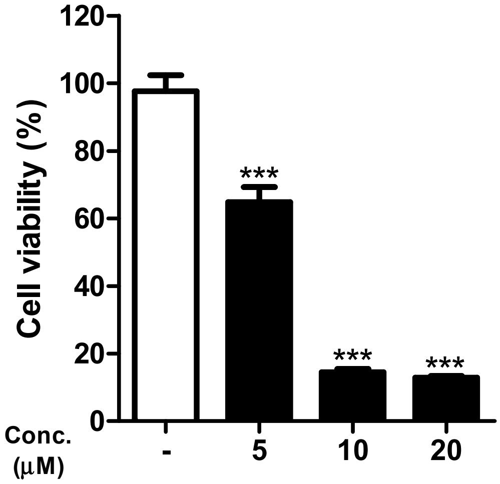

Figure S50. The cytotoxicity effects of compound **10** at different concentrations (5, 10 and 20 μM) in 3T3-L1 adipocytes.

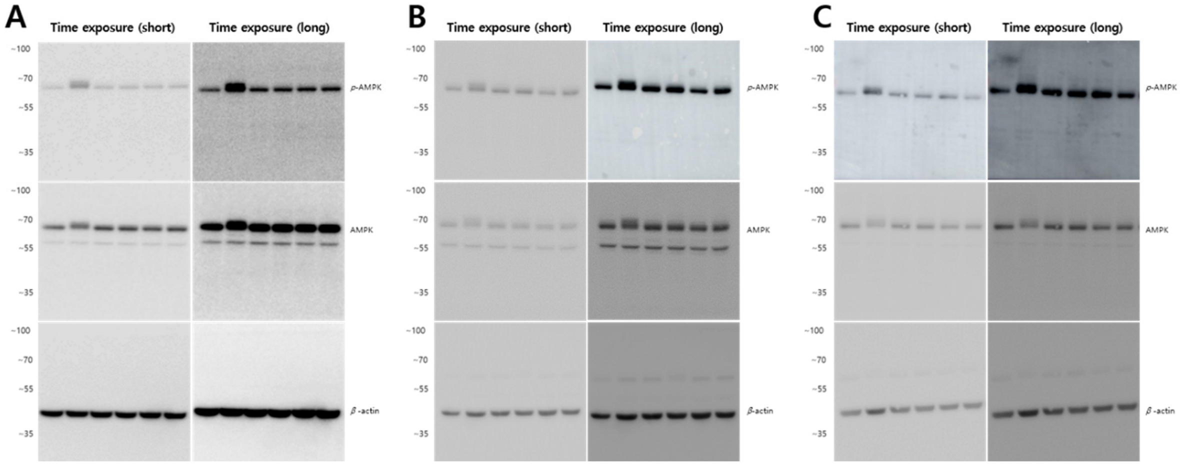

Figure S51. The effects of compounds **3** and **6** on *p*-AMPK (Thr172) in C2C12 cells; original blot images. The expression of *p*-AMPK was firstly observed. The bounding antibodies were removed by using a Restore^{TM} Western blot stripping buffer (Thermo Sci.). The blot was continuously stripped and incubated with AMPK and *β*-actin antibody. Blots from three independent experiments were shown (A-C). Sample names were from left as follows: Ctrl, Aicar (1 mM), compound **3** (10 μM), compound **3** (20 μM), compound **6** (10 μM), and compound **6** (20 μM). *$p < 0.05$, **$p < 0.01$, and ***$p < 0.001$, compared to negative control.

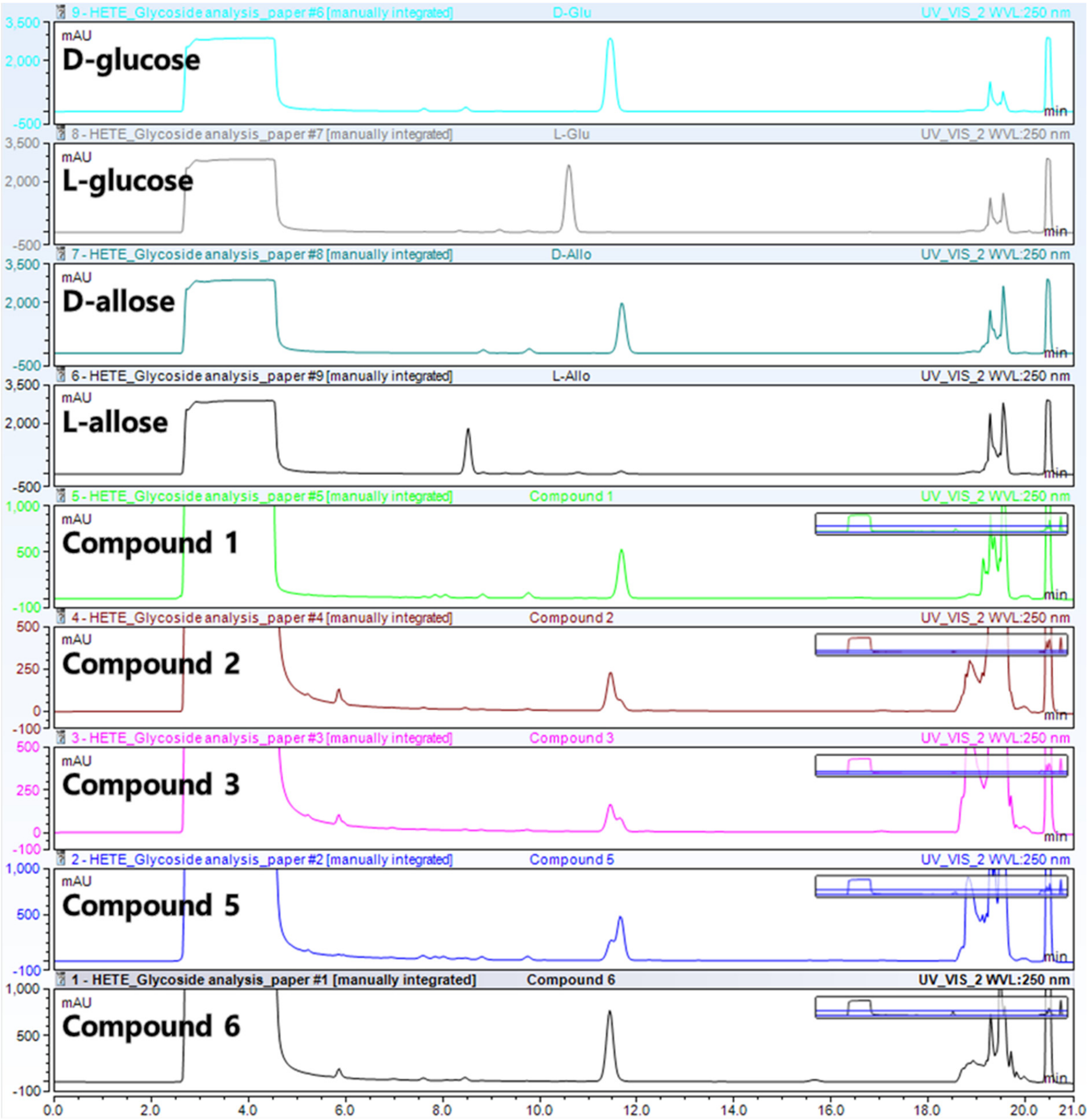

HPLC chromatogram of chiral derivatives from compounds 1−3, 5 and 6 and authentic samples.
